# A Diffusion Approximation and Numerical Methods for Adaptive Neuron Models with Stochastic Inputs

**DOI:** 10.3389/fncom.2016.00039

**Published:** 2016-04-22

**Authors:** Robert Rosenbaum

**Affiliations:** Applied and Computational Mathematics and Statistics, University of Notre DameNotre Dame, IN, USA

**Keywords:** stochastic modeling, Fokker-Planck equation, diffusion approximation, linear response, numerical analysis, spike frequency adaptation

## Abstract

Characterizing the spiking statistics of neurons receiving noisy synaptic input is a central problem in computational neuroscience. Monte Carlo approaches to this problem are computationally expensive and often fail to provide mechanistic insight. Thus, the field has seen the development of mathematical and numerical approaches, often relying on a Fokker-Planck formalism. These approaches force a compromise between biological realism, accuracy and computational efficiency. In this article we develop an extension of existing diffusion approximations to more accurately approximate the response of neurons with adaptation currents and noisy synaptic currents. The implementation refines existing numerical schemes for solving the associated Fokker-Planck equations to improve computationally efficiency and accuracy. Computer code implementing the developed algorithms is made available to the public.

## 1. Introduction

Linking neuronal membrane dynamics, synaptic input statistics and spike train statistics is a central problem in computational neuroscience. The forward problem of mapping membrane dynamics and input statistics to spiking statistics, such as firing rates, is important for understanding how biological neurons transmit sensory and motor signals. The inverse problem of mapping spike train statistics to input statistics and membrane dynamics is critical for inferring synaptic connectivity and neuron properties from extracellular recordings (Pillow et al., 2008; Pernice and Rotter, 2013). Efficient and accurate solutions to both the forward and backward problem rely on computational models of neurons that balance simplicity and computational tractability with biological realism. The adaptive exponential integrate-and-fire (AdEx) neuron model has been proposed as a balance between these needs: It is simple enough to be amenable to mathematical analysis, computationally efficient to simulate and its parameters can be fit to accurately capture the responses of diverse types of biological neurons (Fourcaud-Trocme et al., 2003; Richardson et al., 2003; Brette and Gerstner, 2005; Jolivet et al., 2008; Naud et al., 2008; Touboul and Brette, 2008; Richardson, 2009; Hertäg et al., 2014; Ocker and Doiron, 2014).

Noise is pervasive in the brain. Unreliable neurotransmitter release, ion channel noise and irregular arrival of synaptic inputs combine so that spiking activity is highly irregular with mean spike counts approximately equal to their variance (Softky and Koch, 1993; Shadlen and Newsome, 1994, 1998; Faisal et al., 2008). While the deterministic dynamics of the AdEx model are well-characterized (Naud et al., 2008; Touboul and Brette, 2008), its response to the strong noise observed in cortical circuits is more difficult to analyze mathematically. Spike train statistics can be approximated from Monte-Carlo simulations, but it is computationally expensive to obtain accurate approximations. Fokker-Planck techniques offer an alternative method for approximating spiking and membrane potential statistics. The threshold integration method (Richardson, 2007, 2008; Richardson and Swarbrick, 2010) provides efficient numerical schemes for solving Fokker-Planck equations associated with integrate-and-fire neuron models, but is only applicable when the Fokker-Planck equation is one-dimensional. Temporally correlated inputs and adaptation currents introduce additional dimensions that prevent the direct application of threshold integration. This shortcoming is partially overcome by approximations that reduce the dimensionality of the Fokker-Planck equations: A quasi-static approximation can be used to capture the effects of slow and weak adaptation with one dimension (Richardson, 2009; Ocker and Doiron, 2014). Matched variance approximations capture the effects of temporally correlated synaptic inputs in one dimension by scaling the noise coefficient to capture passive membrane variability (Alijani and Richardson, 2011; Hertäg et al., 2014).

In this article, we present a suite of numerical algorithms for solving Fokker-Planck equations associated with the AdEx model driven by stochastic synaptic input. We extend the matched variance approach (Alijani and Richardson, 2011; Hertäg et al., 2014) to account for the membrane potential fluctuations introduced by a voltage-based adaptation current and to account for a wider class of temporally correlated input. This approximation more accurately captures the effects of strong or fast voltage-based adaptation on neuronal response statistics than previous approximations for adaptive neuron models (Richardson, 2009; Ocker and Doiron, 2014). Moreover, we developed a higher order solver for the numerical implementation of the threshold-integration scheme (Richardson, 2007, 2008) to solve the associated Fokker-Planck equations more efficiently. All of these numerical schemes are combined into user-friendly computer code that is publicly available at http://www.mathworks.com/matlabcentral/fileexchange/55337-adex-threshold-integrator.

## 2. Methods

### 2.1. Model description

We consider integrate-and-fire model neurons with linear adaptation, defined by Brette and Gerstner (2005)

(1) $$C_{m}\frac{dV}{dt}=g_{L}(E_{L}-V)+\psi (V)-w+I$$

(2) $$\tau_{w}\frac{dw}{dt}=-w+a(V-E_{L})$$

(3) $$V\geq V_{\text{th}}\Rightarrow V\to V_{\text{re}}\text{ and }w\to w+b$$

where Equation (3) indicates that each time *V*(*t*) reaches a threshold at *V*_*th*_, it is reset to *V*_*re*_ and *w*(*t*) is incremented by a fixed amount, *b*. In this expression, *C*_*m*_ is the membrane capacitance, *V*(*t*) is the membrane potential, *g*_*L*_ is a leak conductance, *E*_*L*_ is the leak reversal potential, *w*(*t*) is an adaptation variable with time constant τ_*w*_, *b* captures the spike-dependence of *w*(*t*) and *a* captures the voltage-dependence of *w*(*t*). Our numerical methods are derived for general ψ(*V*), but all simulations use the adaptive exponential integrate-and-fire (AdEx) model (Fourcaud-Trocme et al., 2003; Brette and Gerstner, 2005), for which

$$\psi (V)=g_{L}\Delta_{T}e^{(V-V_{T})/\Delta_{T}}.$$

Our methods are also easily adapted to the closely related Izhikevich model, which contain a quadratic, instead of exponential, non-linearity (Izhikevich, 2003).

We develop numerical approximations that can be applied to any stationary stochastic input current, *I*(*t*), but all examples use a model of the form

(4) $$I(t)=J_{\text{e}}\sum_{k}\alpha_{\text{e}}(t-t_{k}^{\text{e}})+J_{\text{i}}\sum_{k}\alpha_{\text{e}}(t-t_{k}^{\text{i}}).$$

Here, *J*_*e*_ > 0 and *J*_*e*_ < 0 are excitatory and inhibitory synaptic weights, α_*x*_(*t*) is a postsynaptic current waveform (EPSC and IPSC), and $t_{k}^{x}$ are spike times for *x* = e, i. Without loss of generality, assume that ∫α_*x*_(*t*)*dt* = 1. We consider two instantiations of this model, one is temporally uncorrelated and the other temporally correlated.

The temporally uncorrelated input model is achieved by letting excitatory and inhibitory spike arrive as homogeneous Poisson processes with rates *r*_*e*_ and *r*_*i*_, and by taking the synaptic currents to be Dirac delta functions,

$$\alpha_{x}(t)=\delta (t),\;x=\text{e},\text{i}.$$

The temporally correlated input model is defined by introducing temporal correlations to the spike times and to the synaptic kinetics. Excitatory and inhibitory spike times arrive as inhomogeneous Poisson processes. The time-dependent firing rates, ν_*x*_(*t*), obey Ornstein-Uhlenbeck dynamics,

$$\tau_{\nu }\frac{d\nu_{x}}{dt}=-\nu_{x}+r_{x}+\sigma_{\nu }\sqrt{2\tau_{\nu }}\eta_{x}(t),$$

for *x* = *e, i* where τ_ν_ sets the timescale of firing rate fluctuations, *r*_*x*_ is the stationary mean rate, σ_ν_ is the stationary standard deviation and η_*x*_(*t*) is standard Gaussian white noise. As long as σ_ν_ ≪ *r*_*x*_, firing rates are positive with overwhelmingly large probability (Merkel and Lindner, 2010; Rosenbaum et al., 2012b). Synaptic kinetics for the temporally correlated input model are captured by setting (Dayan and Abbott, 2001)

$$\begin{array}{l} \alpha_{\text{e}}(t)=\frac{J_{\text{e}}}{\tau_{\text{e}}}e^{-t/\tau_{\text{e}}}\Theta (t)\\ \alpha_{\text{i}}(t)=\frac{J_{\text{i}}}{\tau_{d,\text{i}}-\tau_{r,\text{i}}}(e^{-t/\tau_{d,\text{i}}}-e^{-t/\tau_{r,\text{i}}})\Theta (t) \end{array}$$

where Θ(*t*) is the Heaviside step function, τ_*e*_ is the decay timescale of EPSCs and τ_*r, i*_ < τ_*d, i*_ are the rise and decay timescales of IPSCs respectively. Synaptic time scales were chosen to mimic the kinetics of AMPA and GABA_*B*_ mediated kinetics (see Table 1 and Dayan and Abbott, 2001).

**Table 1:** **Default parameter values**.

τ_*m*_ (ms)	15
*E*_*L*_ (mV)	−72
Δ_*T*_ (mV)	1
*V*_*T*_ (mV)	−55
*V*_*th*_ (mV)	−45
*V*_*re*_ (mV)	−72
*a*∕*C*_*m*_ (kHz)	0.15
*b*∕*C*_*m*_ (mV·kHz)	0.025
τ_*w*_ (ms)	50
*J*_*e*_, *J*_*i*_ (mV)	0.4, −0.75
*r*_*e*_, *r*_*i*_ (kHz)	10, 2
τ_*e*_ (ms)	4
τ_*d, i*_ (ms)	6
τ_*r, i*_ (ms)	1
τ_ν_ (ms)	10
σ_ν_ (Hz)	100

*Default model parameters used in all figures unless otherwise specified in figure caption. Note that the precise values of C_m_ and g_L_ do not need to be specified since the dynamics only depend on their ratio, τ_m_ = C_m_∕g_L_. Note also that the last five parameters are only relevant for the temporally correlated input model*.

We consider the neuron response statistics and the accuracy of our approximations under a variety of different parameter values. The “default” parameter values, used in all simulations except where explicitly stated otherwise in figure captions and axes labels, are given in Table 1.

For all Monte Carlo simulations, Equations (1–3) were solved using a forward Euler scheme with a time bin size of *dt* = 0.1 ms, except where specified otherwise. Each Monte Carlo simulation was of length 65 s. The first 5 s of all simulations were not used in computing statistics, so exactly 60 s were used. Statistics were computed by averaging over 10 such Monte Carlo simulations, except where specified otherwise. All simulations and numerical computations were performed on a MacBook Pro running OS X 10.11.2 with a 2.3 GHz Intel Core i7 processor and 16 GB of 1600 MHz DDR3 RAM. Simulations were run in Matlab R2015b (Mathworks) using the mex environment to compile C code that can be run from the Matlab command line.

### 2.2. A review of spectral and statistical measures of stationary stochastic processes

We are interested in the steady-state statistics of membrane potential and spiking activity when the input current, *I*(*t*), is modeled as a stationary stochastic process. We first briefly review the statistical measures of stationary stochastic processes used in this study. A more in depth treatment can be found elsewhere (Yaglom, 2004; Tetzlaff et al., 2008). We model the spike train of a neuron as a sum of Dirac delta functions,

$$s(t)=\sum_{k}\delta (t-t_{k})$$

where *t*_*k*_ is the *k*-th threshold crossing of *V*(*t*). The steady-state firing rate is given by

$$r_{0}=\underset{T\to \infty }{\lim}T^{-1}\int_{0}^{T}s(t)dt.$$

A common measure of covariability between two processes is the cross-covariance function, defined by

$$C_{XY}(\tau )=\text{cov}(X(t),Y(t+\tau ))$$

for stationary stochastic processes, *X*(*t*) and *Y*(*t*). Analytical computations are often simplified by transitioning to the Fourier domain, defining the cross-spectral density,

$${\tilde{C}}_{XY}(f)=\int_{-\infty }^{\infty }C_{XY}(\tau )e^{-2\pi if\tau }d\tau$$

and the power spectral density, ${\tilde{C}}_{XX}\left(f\right)$. The analytical and numerical computation of cross-spectral densities is simplified by the relationship (Yaglom, 2004),

$${\tilde{C}}_{XY}(f)=\underset{T\to \infty }{\lim}{\tilde{X}}_{T}^{*}(f)\;{\tilde{Y}}_{T}(f)$$

where ^*^ denotes the complex conjugate,

(5) $${\tilde{X}}_{T}(f)=\frac{1}{\sqrt{2T}}\int_{-T}^{T}[X(t)-\mu_{X}]e^{-2\pi ift}dt$$

is the normalized, finite-time Fourier transform of *X*(*t*) with its steady-state mean, μ_*X*_, subtracted and similarly for *Y*_*T*_(*t*). This relationship is particularly useful because it allows convolutions to be treated easily. In particular, suppose that *U*(*t*) = (K*X)(*t*) where * denotes convolution and *K*(*t*) is a deterministic kernel with finite *L*^2^ norm. Then

(6) $${\tilde{U}}_{T}(f)=\tilde{K}(f){\tilde{X}}_{T}(f)$$

for sufficiently large *T* where

$$\tilde{K}(f)=\int_{-\infty }^{\infty }K(t)e^{-2\pi ift}dt$$

is the Fourier transform of *K*(*t*), and therefore

(7) $${\tilde{C}}_{UU}(f)=|\tilde{K}(f){|}^{2}\;{\tilde{C}}_{XX}(f).$$

Finally, the steady-state variance can be computed from the integral of the power-spectral density,

(8) $$\text{var}(X)=\int_{-\infty }^{\infty }{\tilde{C}}_{XX}(f)df$$

These relationships will be helpful in computing several statistics used below.

## 3. Results

### 3.1. A review of the quasi-static approximation for stochastic neuron models with adaptation currents

We first consider the AdEx model from Equations (1–3) with temporally uncorrelated inputs. In this case, a classic diffusion approximation (Gluss, 1967; Capocelli and Ricciardi, 1971; Ricciardi and Sacerdote, 1979) replaces *I*(*t*) from Equations (4) with

(9) $$I(t)=\mu_{I}+\sqrt{2D_{I}}\eta (t)$$

where μ_*I*_ = *J*_*e*_*r*_*e*_ + *J*_*i*_*r*_*i*_ is the mean input bias,

(10) $$D_{I}=\frac{J_{\text{e}}^{2}r_{\text{e}}+J_{\text{i}}^{2}r_{\text{i}}}{2}$$

is the effective diffusion coefficient and η(*t*) is standard Gaussian white noise. This choice of coefficients, μ_*I*_ and *D*_*I*_, assures that the steady-state mean of the input current is captured exactly by the input model and also that the first two infinitesimal moments of the membrane potential are the same for the diffusion approximation as they are for the temporally uncorrelated input model (Gluss, 1967; Capocelli and Ricciardi, 1971; Ricciardi and Sacerdote, 1979).

Under the diffusion approximation in Equations (9), the AdEx model from Equations (1–3) represents a coupled, two-dimensional system of stochastic differential equations. Therefore, the joint probability density of *V* and *w* obeys a two-dimensional Fokker-Planck equation (see Hertäg et al., 2014 for full formulation of the two-dimensional Fokker-Planck equation). In principle, this equation could be solved numerically and the resulting bivariate probability density could be used to compute the firing rate of the neuron. However, the Fokker-Planck equation is computationally expensive and difficult to solve, in part because it has two spatial dimensions and in part because of the non-local boundary conditions associated with the threshold-reset condition in Equation (3) (Richardson, 2009; Hertäg et al., 2014; Ocker and Doiron, 2014).

Previous studies of stochastic neuron models with voltage-activated adaptation currents (Richardson, 2009; Hertäg et al., 2014; Ocker and Doiron, 2014) resolve the difficulty inherent in solving a two-dimensional equation by utilizing the fact that the adaptation time constant, τ_*w*_, is typically much larger than the membrane time constant, τ_*w*_ ≫ τ_*m*_ = *C*_*m*_∕*g*_*L*_. This separation of timescales justifies a quasi-static approximation in which *w* in Equation (1) is replaced by its steady-state mean value, μ_*w*_. Under this approximation, the AdEx model can be viewed as a non-adaptive exponential integrate-and-fire model (EIF, obtained by setting *a* = *b* = 0) with an extra bias term accounting for the mean adaptation current. The only difficulty is that the steady-state mean adaptation current depends on the steady-state firing rate and steady state mean membrane potential, and vice versa. In Richardson (2009) and Ocker and Doiron (2014), this difficulty was overcome by numerically computing the fixed point of their dependence as described next.

The linearity of Equation (2) allows the steady-state mean value of *w*(*t*) to be computed exactly in terms of the firing rate and the steady state mean of *V*(*t*). Specifically, taking expectations in Equation (2) and including the spike-based perturbations from Equation (3) gives,

(11) $$\tau_{w}\frac{d\langle w\rangle }{dt}=a(\langle V\rangle -E_{L})-\langle w\rangle +\tau_{w}br$$

where 〈*w*〉 is the time-dependent mean of *w*(*t*), 〈*V*〉 is the time-dependent mean membrane potential and *r*(*t*) is the instantaneous firing rate of the neuron. The last term in this equation captures the fact that *w*(*t*) is incremented by *b* each time the neuron spikes, as indicated in Equation (3). The steady-state mean is given by finding the unique fixed point of this differential equation,

(12) $$\begin{array}{l} \mu_{w}=\underset{t\to \infty }{\lim}\langle w\rangle \\ \;=a(\mu_{V}-E_{L})+\tau_{w}br_{0} \end{array}$$

where μ_*V*_ and *r*_0_ are the steady-state mean membrane potential and firing rate respectively.

Once *w*(*t*) is replaced by its steady-state mean value and *I*(*t*) is replaced by Gaussian white noise plus a bias term, μ_*I*_, Equation (1) is a one-dimensional stochastic differential equation,

(13) $$\begin{array}{l} C_{m}\frac{dV}{dt}=\;g_{L}(E_{L}-V)+\psi (V)\\ \;-\mu_{w}+\mu_{I}+\sqrt{2D_{I}}\eta (t),\\ V\geq V_{\text{th}}\Rightarrow V\to V_{\text{re}}. \end{array}$$

Thus, the steady-state probability density of *V* and the steady state firing rate can be computed by solving a time-independent Fokker-Planck equation, parameterized by μ_*w*_ (see Appendix A for precise formulation of the one-dimensional Fokker-Planck equation).

This Fokker-Planck equation can be solved numerically efficiently using the threshold integration method developed in Richardson (2007, 2008). We improved computational efficiency of previous implementations of the threshold integration method in several ways. First, we implemented the solver in the C programming language. To maintain usability, we used the Matlab “mex” environment so that the compiled C code can be called from the Matlab command line. Additionally, solver allows for a variable mesh size so that the mesh can be refined where the derivative of the solution is expected to be large in magnitude.

Finally, we improved the numerical scheme used to implement the threshold-integration method. Previous implementations used a “modified Euler scheme” that exploits the linearity of the Fokker-Planck equation to write the solution in terms of integrals over mesh elements (Richardson, 2008). Whereas these previous implementations used the midpoint rule to approximate the resulting integrals, we used the more accurate Simpson's rule. A detailed description of the numerical solver is given in Appendix A. The performance of our implementation is compared to previous implementations below.

In summary, the mean membrane potential and firing rate, μ_*V*_ and *r*_0_, can be computed as a function of the mean adaptation current, μ_*w*_, by numerically solving the Fokker-Planck equation. Similarly, the steady-state mean of the adaptation variable can be computed in terms of the steady-state mean membrane potential and firing rate using Equation (12). Combining these two strategies, the steady state statistics can be approximated using fixed point iteration (see Appendix B). We hereafter refer to the approximation obtained this way as the “quasi-static approximation” to steady-state statistics. This approach was used previously in Richardson (2009) and Ocker and Doiron (2014).

**Figure 1:**
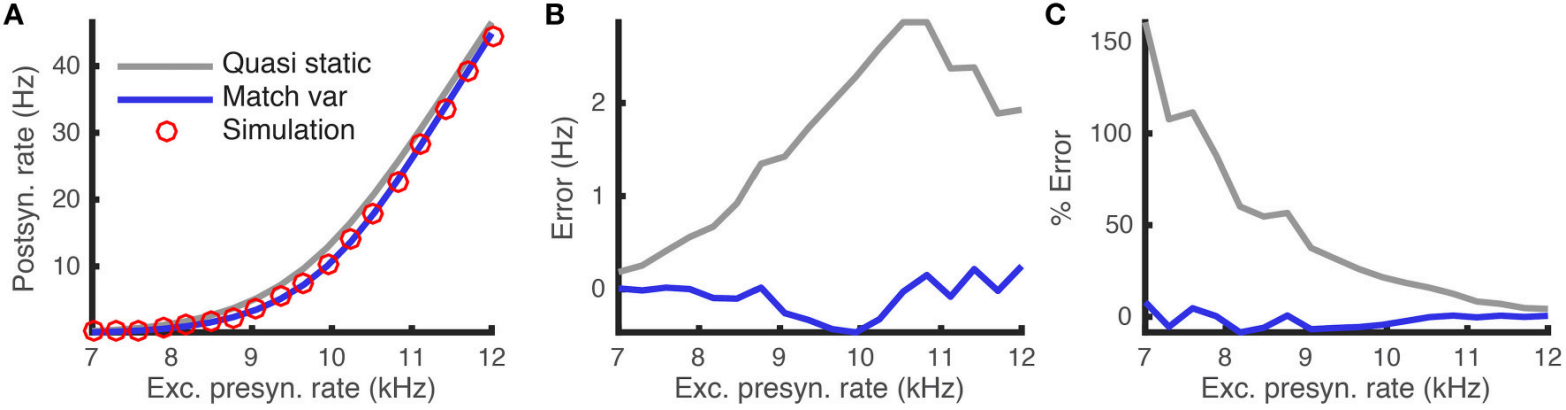
**Quasi-static and matched variance approximations to the firing rate of an adaptive neuron model compared to Monte Carlo simulations as a function of presynaptic input rate**. **(A)** Steady-state postsynaptic Firing rate, *r*_0_, as the presynaptic excitatory rate, *r*_*e*_, increases. Plotted for Monte-Carlo simulations (red circles), quasi-static approximation (gray curve) and matched variance approximation (blue curve). **(B,C)** Error and % error of the quasi-static and matched variance approximations compared to Monte-Carlo simulations. The temporally uncorrelated input model was used with all parameters except *r*_*e*_ given in Table 1.

The quasi-static approximation is expected to be accurate when adaptation is slow and weak (Richardson, 2009; Ocker and Doiron, 2014). We tested the accuracy of the quasi-static approximation with stronger and fast adaptation (see Methods) as a function of the the rate, *r*_*e*_, of excitatory presynaptic spikes (Figure 1). While the quasi-static approximation captured the overall shape of the dependence of steady state firing rate on *r*_*e*_ (Figure 1A, compare red circles and gray curve), the errors were substantial in magnitude and percentage (Figures 1B,C, gray curve).

We next show that these errors are due largely to the fact that the quasi-static approximation does not account for sub-threshold, passive membrane potential variability elicited by sub-threshold, voltage-based adaptation. We then propose an improved approximation that accounts for this variability.

### 3.2. A matched variance approximation to sub-threshold adaptation dynamics

We conjectured that the errors made by the quasi-static approximation were largely due sub-threshold fluctuations in *w*(*t*), which are not accounted for by the quasi-static approximation. Our goal in this section is to derive a diffusion approximation that accurately captures the steady-state free membrane potential variance when sub-threshold adaptation is taken into account.

To demonstrate the source of the errors made by the quasi-static approximation, first consider the free membrane potential under the quasi-static approximation, defined by removing active currents and spiking from Equation (13) to obtain

$$\begin{array}{l} C_{m}\frac{dU}{dt}=g_{L}(E_{L}-U)-\mu_{w}\\ \;-\mu_{I}+\sqrt{2D_{I}}\eta (t). \end{array}$$

This represents an Ornstein-Uhlenbeck process with steady-state variance (Gardiner, 1985)

(14) $$\text{var}(U)=\frac{D_{I}\tau_{m}}{C_{m}^{2}}.$$

where τ_*m*_ = *C*_*m*_∕*g*_*L*_. Thus, the variance of the free membrane potential under the quasi-static approximation does not depend on adaptation dynamics at all (since Equation 14 does not depend on μ_*w*_, τ_*w*_, *a* or *b*).

For the full AdEx model, the sub-threshold membrane potential is affected by fluctuations in *w*(*t*) through voltage-based adaptation. This can be seen by considering the free membrane potential and adaptation dynamics defined by omitting spike-activation and spiking dynamics from Equations (1–3) to obtain

(15) $$\begin{array}{l} C_{m}\frac{dU}{dt}=-g_{L}(U-E_{L})-W+I\\ \tau_{w}\frac{dW}{dt}=-W+a(U-E_{L}). \end{array}$$

Here, *U*(*t*) represents the free membrane potential and *W*(*t*) the “free adaptation” current. Since these equations are coupled, fluctuations in *W*(*t*) affect fluctuations in *V*(*t*) and vice versa.

In Appendix C, we show that the steady-state variance of *U*(*t*) under Equations (15) for the temporally uncorrelated input model is given by

(16) $$\text{var}(U)=\frac{D_{I}\tau_{m}}{C_{m}^{2}}\left[1-\left(\frac{a}{a+g_{L}}\right)\left(\frac{\tau_{m}}{\tau_{m}+\tau_{w}}\right)\right]$$

where $D_{I}=\left(J_{\text{e}}^{2}r_{\text{e}}\;+\;J_{\text{i}}^{2}r_{\text{i}}\right)∕2$ as above. This reveals a major source of error in the quasi-static approximation. As noted above, the free membrane potential variance for the quasi-static approximation (Equation 14) does not depend on adaptation at all. On the other hand, the free membrane potential variance of the full model is affected by sub-threshold adaptation currents, since Equation (16) depends on *a* and τ_*w*_. Comparing Equations (14) and (16) reveals that the steady-state variance under the quasi-static approximation is least accurate when adaptation is strong (*a* larger relative to *g*_*L*_) and/or fast (τ_*w*_ smaller relative to τ_*m*_).

**Figure 2:**
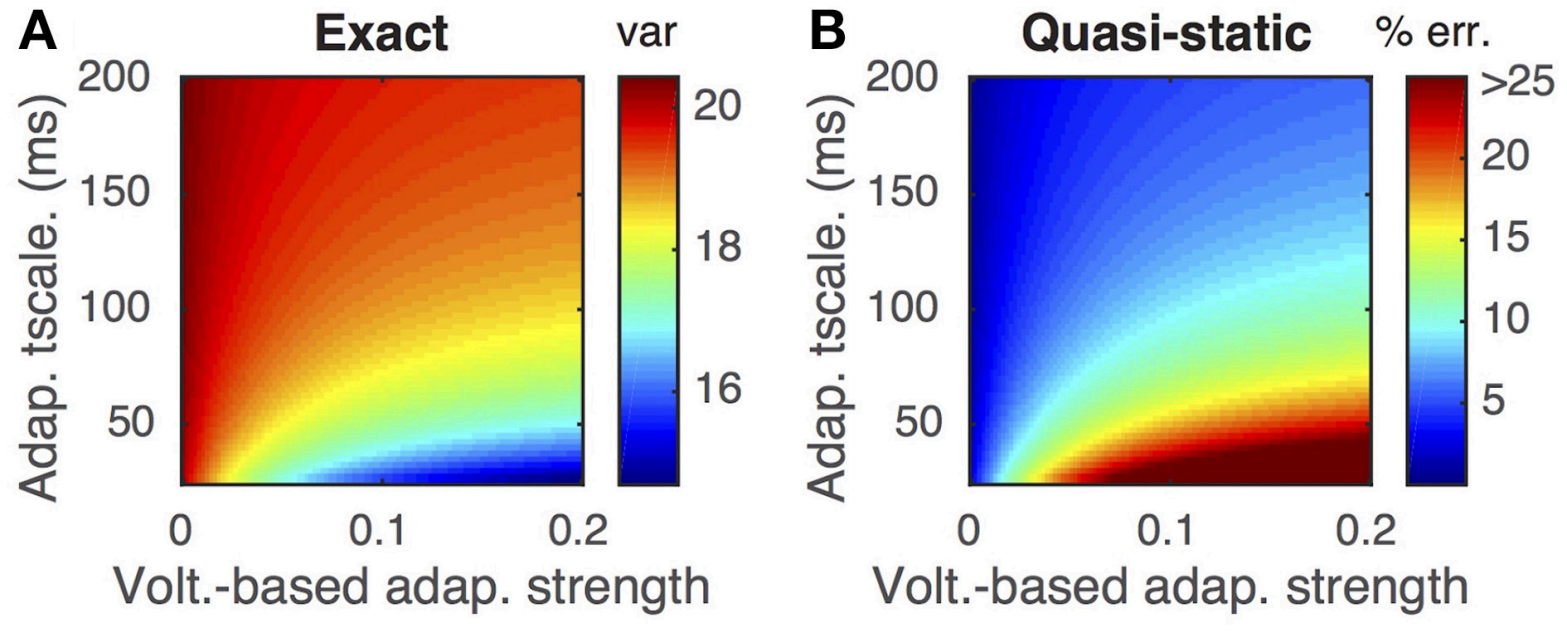
**Steady-state free membrane potential variance and the error in estimating it with a quasi-static approximation**. **(A)** Steady-state free membrane potential variance when sub-threshold adaptation is accounted for, from Equation (16), as a function of voltage-based adaptation strength, *a*, and adaptation timescale, τ_*w*_. **(B)** Percent error of quasi-static approximation to the steady-state membrane potential variance, from Equation (14), vs. the variance from **(A)**. Adaptation strengths, *a*, are reported in units kHz·*C*_*m*_. Voltage variance has units mV^2^. All parameters other than *a* and τ_*w*_ are as in Table 1.

**Figure 3:**
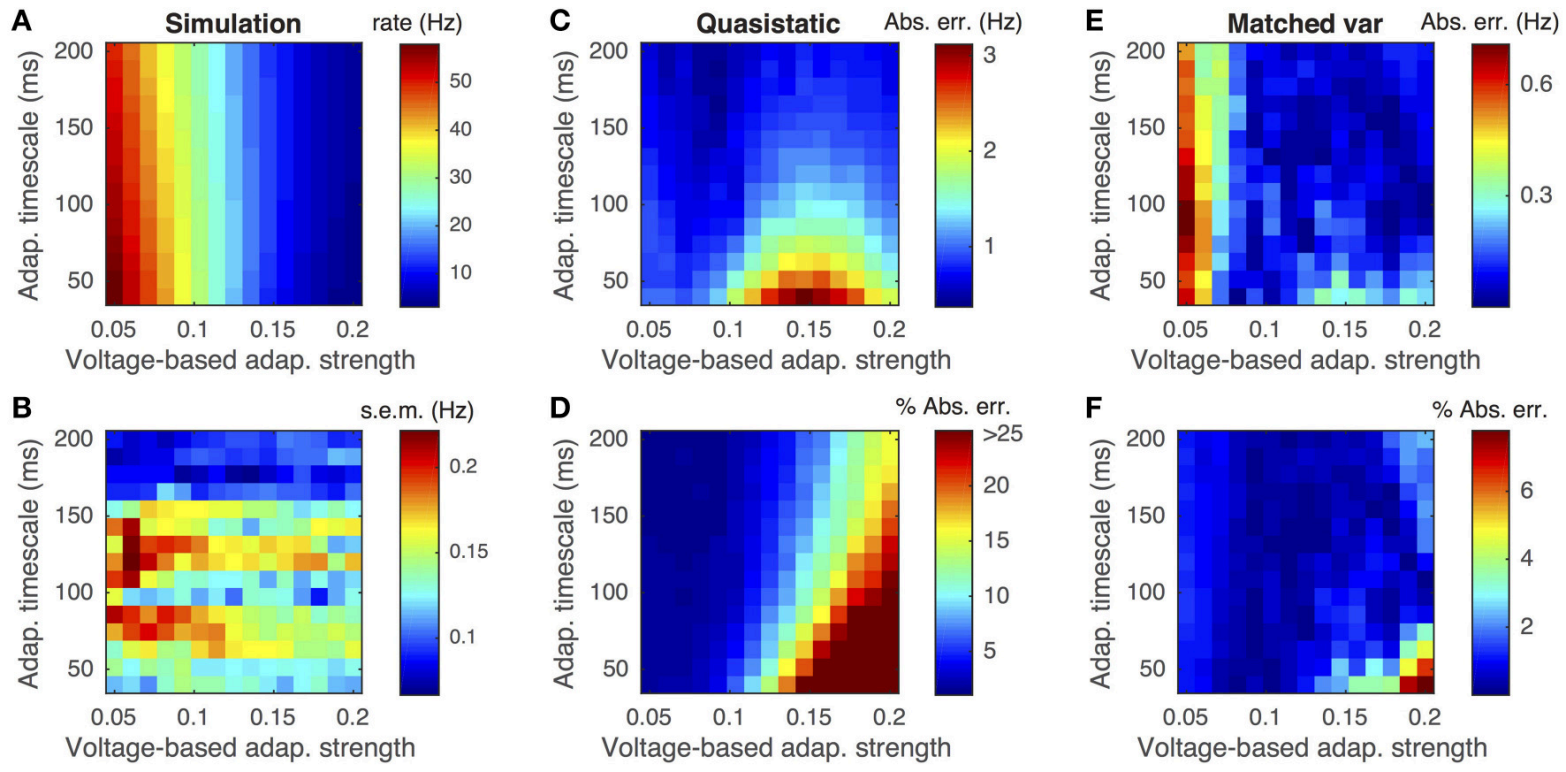
**Firing rate approximations and errors as a function of voltage-based adaptation strength and timescale**. **(A)** Firing rate from Monte-Carlo simulations as a function of voltage-based adaptation strength, *a*, and adaptation timescale, τ_*w*_. **(B)** Standard error of the mean from simulations in **(A)**. **(C,D)** Absolute error and percent absolute error of quasi-static approximation to firing rate compared to simulations from **(A)**. **(E,F)** Same as **(C,D)**, but for matched variance approximation. Adaptation strengths, *a*, are reported in units kHz·*C*_*m*_. The temporally uncorrelated input model was used. All parameters other than *a* and τ_*w*_ are as in Table 1.

This difference is demonstrated by comparing the approximated and actual free membrane potential variance for different values of *a* and τ_*w*_ (Figure 2). The quasi-static approximation does especially poorly in capturing the free membrane potential variability when *a* is large or τ_*w*_ is small (Figure 2B). These errors in approximating the variability of sub-threshold membrane potential variability introduce errors in the resulting firing rate approximation. Notably, the percent error made in approximating the firing rate depends similarly on the adaptation parameters as the percent error made in approximating the membrane potential variance (Figures 3C,D, compare to Figure 2B).

We now derive a one-dimensional “matched variance” diffusion approximation that accurately captures the steady-state free membrane potential variance of the two-dimensional model in Equation (15). This diffusion approximation is defined by

(17) $$\begin{array}{l} C_{m}\frac{dU}{dt}=-g_{L}(U-E_{L})-\mu_{w}+\mu_{I}\\ \;+\sqrt{2D_{\text{eff}}}\eta (t) \end{array}$$

where η(*t*) is standard Gaussian white noise and the diffusion coefficient, *D*_*eff*_, is scaled to account for the variability of *V*(*t*) in the presence of sub-threshold adaptation. The steady-state free membrane potential variance from Equation (17) is Gardiner (1985)

(18) $$\text{var}(U)=\frac{D_{\text{eff}}\tau_{m}}{C_{m}^{2}}.$$

We want to choose *D*_*eff*_ so that this variance is equal to the variance in Equation (16) from the two-dimensional model. This is achieved by setting Equation (16) equal to Equation (18) and solving for *D*_*eff*_ to obtain

(19) $$D_{\text{eff}}=D_{I}\left[1-\left(\frac{a}{a+g_{L}}\right)\left(\frac{\tau_{m}}{\tau_{m}+\tau_{w}}\right)\right]$$

where $D_{I}=\left(J_{\text{e}}^{2}r_{\text{e}}+J_{\text{i}}^{2}r_{\text{i}}\right)∕2$. This choice of *D*_*eff*_ defines the “matched variance” approximation under the temporally uncorrelated input model (see Methods).

In summary, the matched variance diffusion approximation, given by using Equation (17) with *D*_*eff*_ from Equation (19), achieves the same steady state free membrane potential variance as the two-dimensional model in Equation (15) under the temporally uncorrelated input model (see Methods). This approach is similar to previous matched variance approximations that account for the effects of temporally correlated noise, but not sub-threshold adaptation, on the free membrane potential variance (Alijani and Richardson, 2011; Hertäg et al., 2014). Below, we consider a combination of these approaches that accounts for both voltage-based adaptation and temporally correlated inputs.

Once *D*_*eff*_ is computed, the matched variance approximation to the firing rates can be computed using the same Fokker-Planck solver and iterative methods used for the quasi-static approximation reviewed above and in Appendices A and B. The only difference between the quasi-static and matched variance approximations is the choice of diffusion coefficient, *D*_*I*_ or *D*_*eff*_.

The matched variance diffusion approximation corrects a substantial portion of the error made by the quasi-static approximation, especially when the neuron is in the fluctuation dominated regime (Figure 1, compare blue and gray). This is because the fluctuations introduced by the adaptation current alter firing rates the most in the fluctuation driven regime.

Moreover, the matched variance approximation provides a substantially improved firing rate approximation whenever voltage-based adaptation is strong and/or fast (*a* large, τ_*w*_ small; Figures 3E,F). Notably, the absolute error in firing rate is less than 1 Hz and the relative error less than 10% for the entire set of parameters we considered (Figures 3E,F). It was greater than 1 Hz and 10% for a considerable portion of the parameters using the quasi-static approximation (Figures 3C,D). Nevertheless, the matched variance approximation replaces the temporally correlated adaptation current with white noise which introduces some error, especially when adaptation is strong and fast (Figure 3F).

The matched variance approximation offers essentially the same computational efficiency as the quasi-static approximation since they only differ by the value of the diffusion coefficient used. The average CPU time used to compute each firing rate data point in Figure 1 was 1.4 ms for the quasi-static approximation and 1.5 ms for the matched variance approximation.

**Figure 4:**
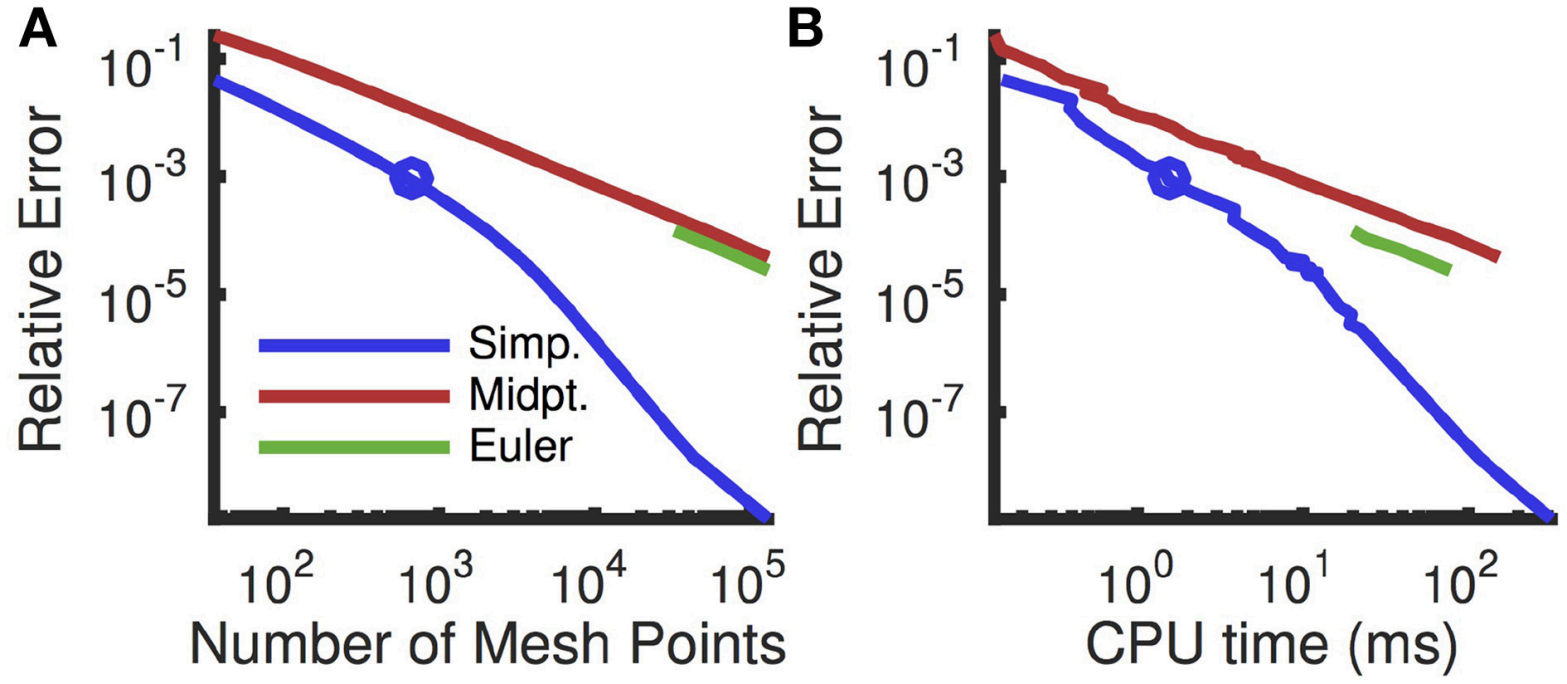
**Convergence of numerical schemes for computing steady state firing rate**. **(A)** Relative numerical error (defined as the relative deviation of the computed firing rate from the rate computed with a fine uniform mesh of size *dv* = 10^−4^ mV which gives 7 × 10^5^ mesh points) plotted as a function of the number of mesh points. The mesh was uniform except near *V*_*re*_ and *V*_*th*_ where it was refined (see Appendix A). **(B)** Same error plotted as a function of CPU time to compute the firing rate. Firing rates were computed using the threshold integration method with a standard Euler solver (green), the modified Euler method from Richardson (2007, 2008) using a midpoint rule for integration (red) and the modified Euler method using Simpson's rule (blue). For the Euler method, the error is not plotted for coarser meshes because they yielded a predicted firing rate of zero. Circle indicates the mesh size used in all other figures. All parameters are as in Table 1.

A comparison of numerical schemes for solving the Fokker-Planck equation shows that the modified Euler scheme with Simpson's rule substantially outperforms the previously proposed modified Euler scheme with the midpoint rule (Figure 4, compare blue and red). Interestingly, a standard Euler scheme slightly outperforms the modified Euler scheme with the midpoint rule (Figure 4, compare green and red), but only gives a reasonable approximation for very fine meshes, due to the large exponential nonlinearity in ψ(*V*) for *V* near *V*_*T*_ (Richardson, 2008).

Despite the fact that the matched variance approximation captures some of the variability introduced by sub-threshold voltage-based adaptation, errors are introduced by several assumptions made by the model. We next explore these other sources of error by modulating various parameters of the model.

**Figure 5:**
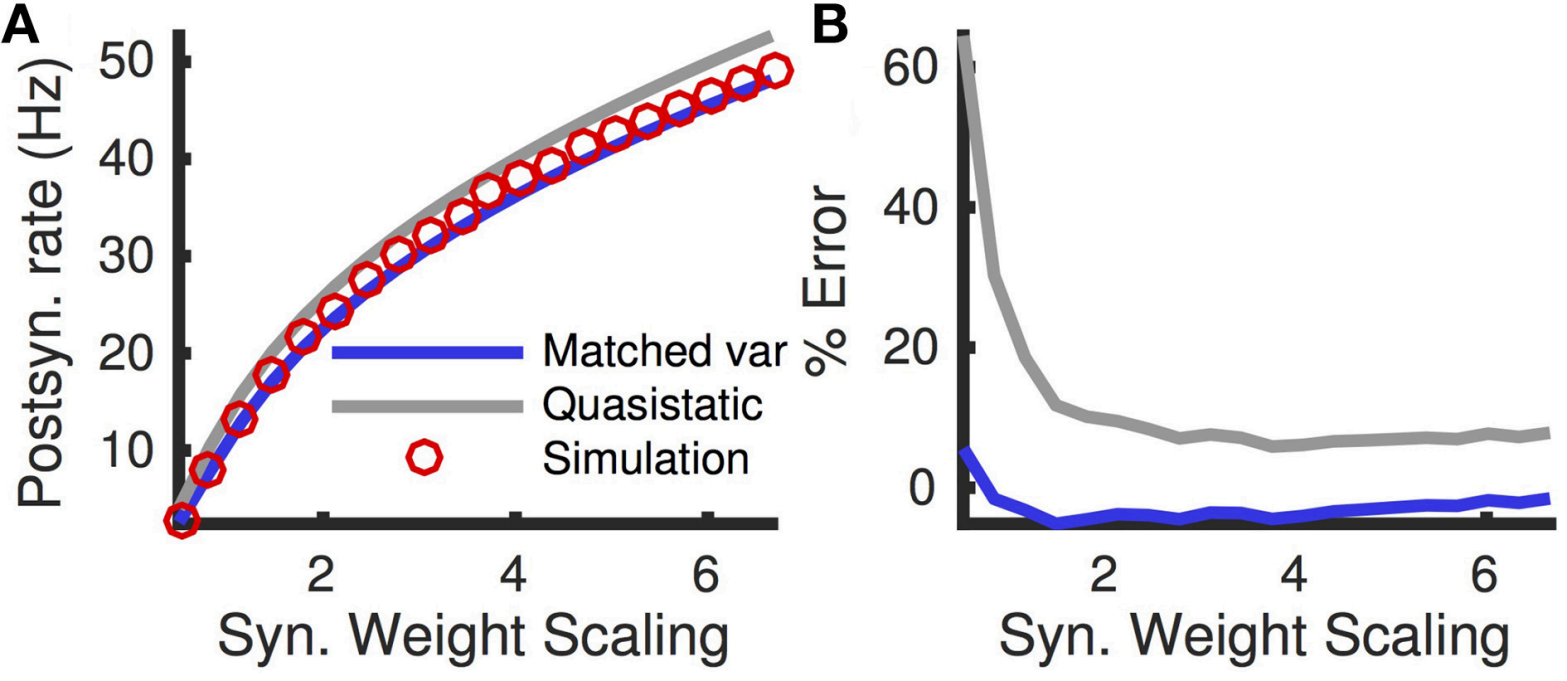
**Firing rate and error as a function of synaptic weights**. **(A)** Steady state firing rate from simulations (red circles), quasi-static approximation (gray) and matched variance approximation (blue) as synaptic weights, *J*_*e*_ and *J*_*i*_, are scaled by different factors. **(B)** Percent errors from **(A)**. Presynaptic firing rates, *r*_*e*_ and *r*_*i*_, were scaled inversely so that μ_*I*_ = *J*_*e*_*r*_*e*_+*J*_*i*_*r*_*i*_ remained constant. The temporally uncorrelated input model was used. All parameters other than *J*_*e*_, *J*_*i*_, *r*_*e*_ and *r*_*i*_ are as in Table 1.

Both the quasi-static and matched variance approximations approximate spike-based inputs with Gaussian white noise. This diffusion approximation is only mathematically valid in the limit of small synaptic weights, *J*_*e*_, *J*_*i*_ ≪ 1, and large presynaptic firing rates, *r*_*e*_, *r*_*i*_ ≫ 1 (Ricciardi and Sacerdote, 1979). To test the dependence of our numerical approximations on the synaptic weights, we computed the error in approximating postsynaptic firing rates as the synaptic weights were scaled and firing rates were scaled inversely, so that the mean input, μ_*I*_ = *J*_*e*_*r*_*e*_ + *J*_*i*_*r*_*i*_ remained fixed. Specifically, we set *J*_*x*_ → *cJ*_*x*_ and *r*_*x*_ → *r*_*x*_∕*c* for *x* = *e, i* and for a range of scalings, *c*. We found that the matched variance approximation accurately predicted the firing rate over a large range of scalings (Figure 5 blue curve, absolute error < 5% for all data points) and substantially outperforms the quasi-static approximation (Figure 5, compare blue and gray).

**Figure 6:**
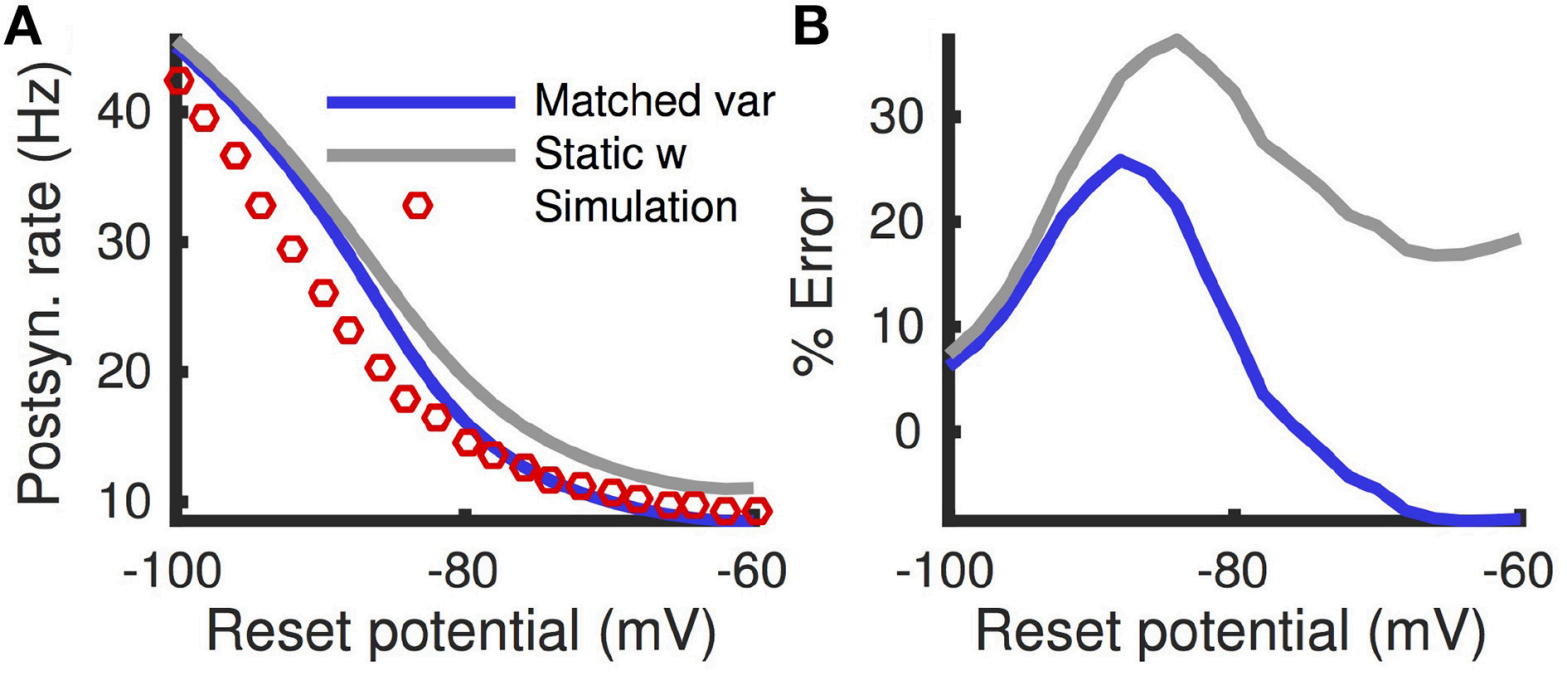
**Firing rate and error as a function of reset potential**. **(A)** Steady state firing rate from simulations (red circles), quasi-static approximation (gray) and matched variance approximation (blue) as a function of the reset potential, *V*_*re*_. **(B)** Percent errors from **(A)**. The temporally uncorrelated input model was used. All parameters other than *V*_*re*_ are as in Table 1.

While the matched variance approximation corrects for some variability introduced by sub-threshold, passive adaptation, it does not correct for fluctuations in the adaptation current evoked by action potentials. Some of this variability is introduced the reset of the membrane potential after a spike, *V* → *V*_*re*_. This reset rule affects the adaptation current, *w*, through the voltage-based adaptation. If *V*_*re*_ is far from the steady-state mean membrane potential, the voltage-reset rule has a larger impact on the adaptation current, so we expect the matched variance approximation to perform poorly since it ignores this impact altogether. Indeed, we found that the matched variance approximation performs especially poorly for *V*_*re*_ < −80 mV (Figure 6). Nevertheless, it still outperforms the quasi-static approximation (Figure 6, compare blue and gray).

Surprisingly, we found that a lower reset potential (*V*_*re*_ more negative) produced a larger firing rate, and this effect was captured by simulations and both numerical approximations (Figure 6A). This is a consequence of strong voltage-based adaptation. Resetting the membrane potential far below *E*_*L*_ also reduces the adaptation current, *w*, through Equation (2). The membrane potential recovers from its reset before *w* (since τ_*m*_ < τ_*w*_), during which time the neuron has an increased likelihood of spiking.

**Figure 7:**
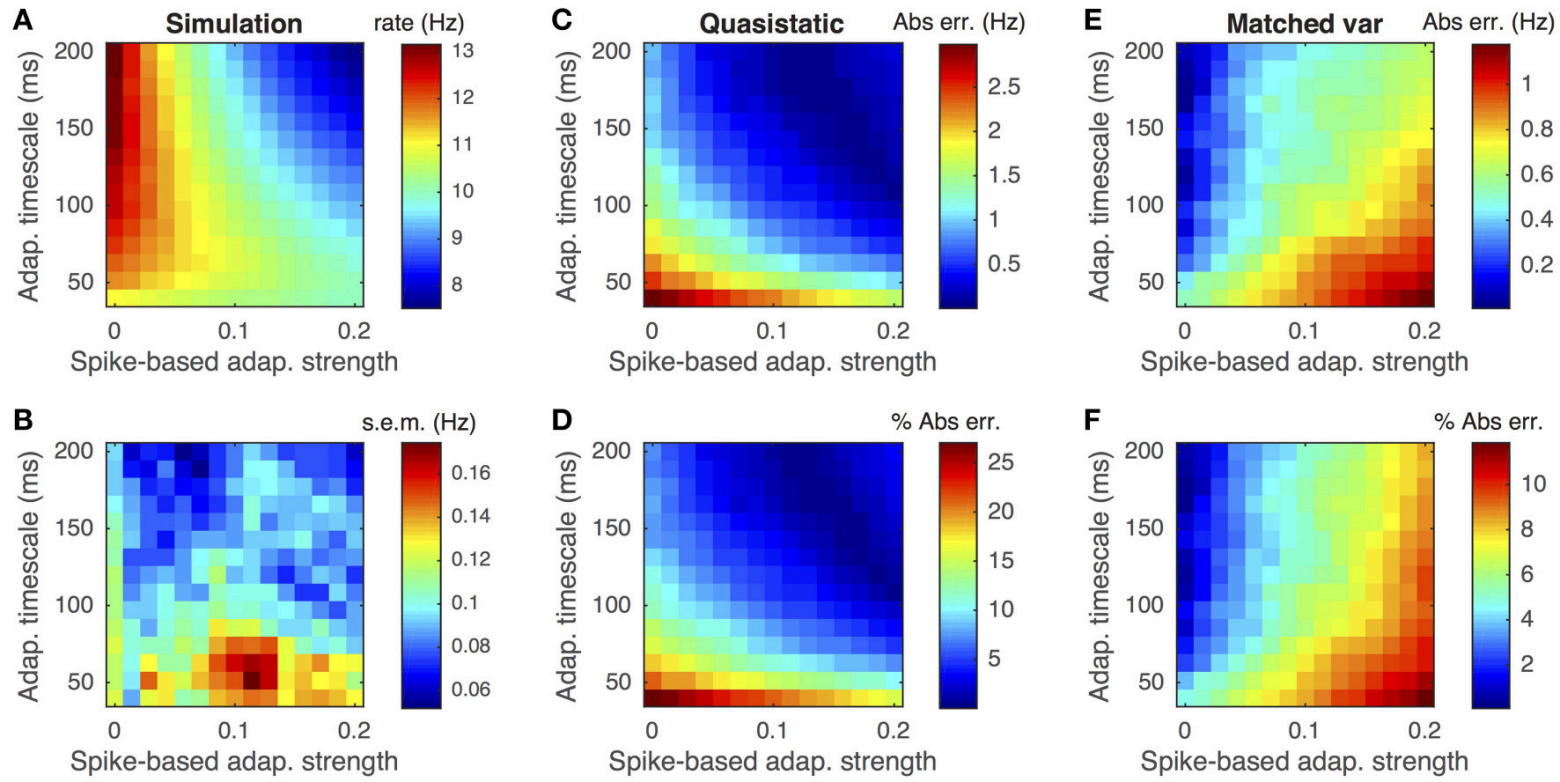
**Firing rate approximations and errors as a function of spike-based adaptation strength and timescale**. **(A–F)** Same as Figures 3A–F except that spike-based adaptation strength, *b*, was varied instead of voltage-based. Adaptation strengths, *b*, are reported in units mV·kHz·*C*_*m*_. The temporally uncorrelated input model was used. All parameters other than *a* and τ_*w*_ are as in Table 1.

While the matched variance approximation captures some sub-threshold fluctuations introduced by voltage-based adaptation, it uses the same approximation to the effects of spike-based adaptation that the quasi-static approximation uses. Thus, we expect both approximations to perform poorly when spike-based adaptation is too strong or fast. We confirmed this expectation by comparing both approximations to Monte-Carlo simulations over a range of spike-based adaptation strengths, *b*, and adaptation timescales, τ_*w*_ (Figure 7). As expected, the matched variance approximation performed most poorly when spike-based adaptation was both fast and strong (Figures 7E,F). The quasi-static approximation performed poorly for faster adaptation, but interestingly performed better for stronger spike-based adaptation (Figures 7C,D). Surprisingly, the quasi-static approximation actually performed better than the matched variance approximation for strong and slow spike-based adaptation.

### 3.3. Numerical methods to compute the linear response to a modulation of input current

So far, we considered numerical methods to compute the steady state firing rate of an adaptive model neuron with stationary inputs. We now consider the response of the neuron to inputs whose statistics exhibit an explicit time-dependence. In general, this would require solving a time-dependent formulation of the Fokker-Planck equation that is not amenable to the threshold-integration methods used for the steady state problem. However, if the temporal modulation of the statistics are weak, threshold-integration methods can be used to solve the time-dependent Fokker-Planck equation to first order in the magnitude of the modulation (Richardson, 2007, 2008). This can be combined with linear response theory to quantify neuronal signal transfer, inter-neuronal correlations and recurrent network stability (Lindner et al., 2005; de la Rocha et al., 2007; Richardson, 2008; Shea-Brown et al., 2008; Ostojic et al., 2009; Ledoux and Brunel, 2011; Trousdale et al., 2012). We follow previously developed methods for computing the response of an adaptive neuron model to a modulation (Richardson, 2009; Ocker and Doiron, 2014), with some refinements. Details of the implementation are given in Appendix D and we review the overall approach here.

In short, we consider a weak perturbation to the synaptic input of the form

$$I(t)=I_{0}(t)+\epsilon u(t)$$

where *I*_0_(*t*) is the un-perturbed input considered above and *u*(*t*) is some stationary perturbation. Linear response theory can be used to show that the finite-time Fourier transform of the firing rate can be written as

$${\tilde{r}}_{T}(f)=\epsilon S_{r}(f){\tilde{u}}_{T}(f)+o(\epsilon )$$

for sufficiently large *T* where ${\tilde{S}}_{r}\left(f\right)$ is the the susceptibility function of the neuron's firing rate (Risken, 1996; Fourcaud and Brunel, 2002).

The susceptibility function can be computed from a one-dimensional Fokker-Planck equation, linearly coupled with an equation for the modulation of the adaptation current. This can in turn be solved using threshold-integration methods. This approach was first developed in Richardson (2009) and also used in Ocker and Doiron (2014). We review the approach and our implementation of it in Appendix D. Similar to our computation of steady-state statistics, we improved the efficiency and accuracy of the susceptibility computation by implementing the solver in C, using Simpson's method to compute integrals and using the matched variance approximation for the effective diffusion coefficient.

It is often necessary to take an inverse Fourier transform of susceptibility functions (see the examples below, for instance). Using Fast Fourier Transform methods for this inverse requires a uniform grid of frequencies. This grid must be fine enough to capture the low-frequencies introduced by adaptation, but must also extend to sufficiently high frequencies to capture faster spiking and membrane potential timescales. As a result, the susceptibility functions must be evaluated at a large number of frequency values. This can be computationally expensive since threshold-integration must be performed separately for each value of *f* (see Appendix D).

**Figure 8:**
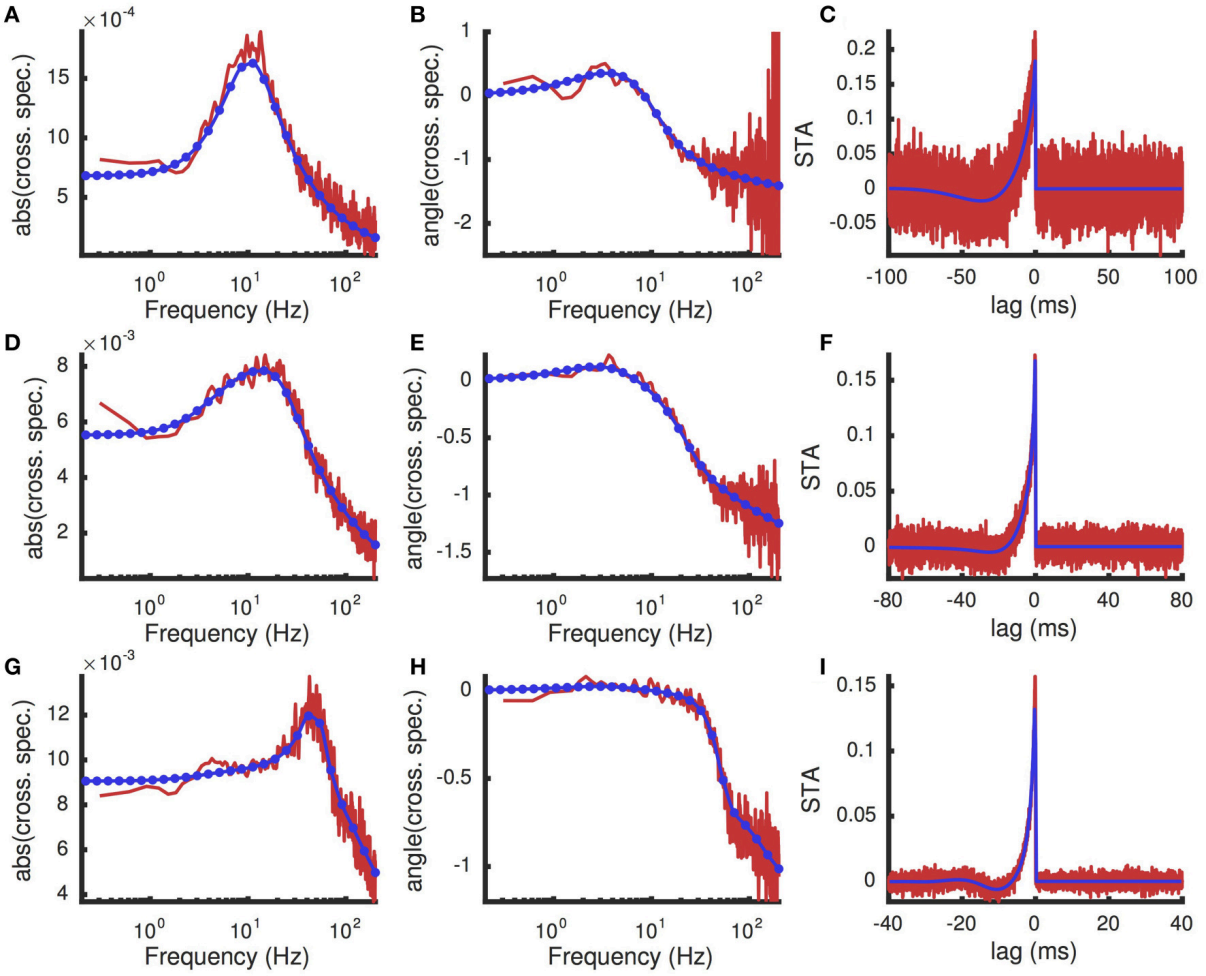
**Spike triggered average input current and cross-spectral density under a white noise perturbation for different excitatory presynaptic rates**. A weak white noise current was added to the model neuron ($D_{n}∕C_{m}^{2}=0.1$(mV)^2^/ms). **(A,B)** The magnitude ($\left|{\tilde{C}}_{us}\right|$ in units *C*_*m*_*mV*) and phase ($\text{angle}\left({\tilde{C}}_{us}\right)$, in radians) of the cross-spectral density between the white noise input and postsynaptic spike train. **(C)** The spike triggered average (units *C*_*m*_*mV*∕*ms*) of the white noise input. **(A–C)** were computed with presynaptic excitatory rate *r*_*e*_ = 8 kHz. **(D–F)** are the same as **(A–C)**, but with *r*_*e*_ = 10 kHz. **(G–I)** are the same, but with *r*_*e*_ = 12 kHz. In all panels, results from Monte Carlo simulations are in red and numerical results from the matched variance approximation are in blue. The filled blue circles in indicate the coarse frequency values at which threshold-integration was applied. The smooth blue curve indicates the interpolated values used to compute the inverse Fourier transforms for the STAs. The temporally uncorrelated input model was used with the additional white noise input. All parameters except *r*_*e*_ are as in Table 1.

To overcome this high computational cost, we note that susceptibility functions of neuron models are typically smooth and slowly varying functions of *f*, especially at higher frequencies and especially when viewed on a log-log scale (see Figure 8, for example). Instead of evaluating them at each value of *f*, we first evaluate them at a coarser, potentially non-uniform mesh of frequencies. Since the functions change more sharply at lower frequencies, we use a mesh that is uniform on a log scale. Once the susceptibility functions are computed on this coarser mesh, interpolation is used to estimate their values on a finer, uniform mesh. The inverse Fast Fourier Transform can then be applied to the interpolated values, since the mesh is uniform and sufficiently fine. We specifically found that using piecewise-cubic Hermite interpolating polynomials (Fritsch and Carlson, 1980) via Matlab's built-in pchip command yields accurate results when the coarser mesh has only 30 points (spaced evenly on a log scale). We next verify this numerical approach by approximating spike triggered averages and inter-neuronal spike train correlations, which require the application of an inverse transform to the susceptibility functions.

### 3.4. Computing spike-triggered average and cross-spectral density between the spike train and an input current perturbation

To test the accuracy of our approximation of the response to a modulation, we performed Monte-Carlo simulations with a weak Gaussian white noise term was added to *I*(*t*),

I(t)→I(t)+u(t)

where

$$u(t)=\sqrt{2D_{n}}\eta_{n}(t)$$

and η_*n*_(*t*) is Gaussian white noise. The excitatory and inhibitory conductances were left un-perturbed for this example.

We considered two ways to quantify the response to the white noise perturbation. We first considered the cross-spectral density, ${\tilde{C}}_{us}\left(f\right)$, between the white noise input and the postsynaptic spike train (see Methods for definition). A direct application of linear response theory shows that the cross-spectral density is given to first order in *D*_*n*_ by

$${\tilde{C}}_{us}(f)=2D_{n}S_{r}(f).$$

Thus, the cross-spectral density can be numerically approximated by computing the susceptibility function. We also considered the spike-triggered average noise, defined by

$$STA(\tau )={\text{avg}}_{j}\left(u(\tau -t_{j})\right)$$

where *t*_*j*_ are the spike times of the neuron. These STA can be computed from the cross-spectral density by first noting that (Dayan and Abbott, 2001)

$$STA(\tau )=\frac{C_{us}(-\tau )}{r_{0}}$$

where *C*_*us*_(τ) = *cov*(*u*(*t*), *s*(*t* + τ)) is the cross-covariance function between the white noise input and the postsynaptic spike train, which is the inverse Fourier transform of ${\tilde{C}}_{us}\left(f\right)$ (see Methods and Tetzlaff et al., 2008). Putting this all together gives

$$STA=\frac{2D_{n}}{r_{0}}{ℱ}^{-1}\left[S_{r}^{*}\right]$$

where F^−1^ denotes the inverse Fourier transform and ^*^ the complex conjugate. Thus, the spike-triggered average can be approximated directly from the susceptibility through an inverse Fourier transform of the susceptibility function.

**Figure 9:**
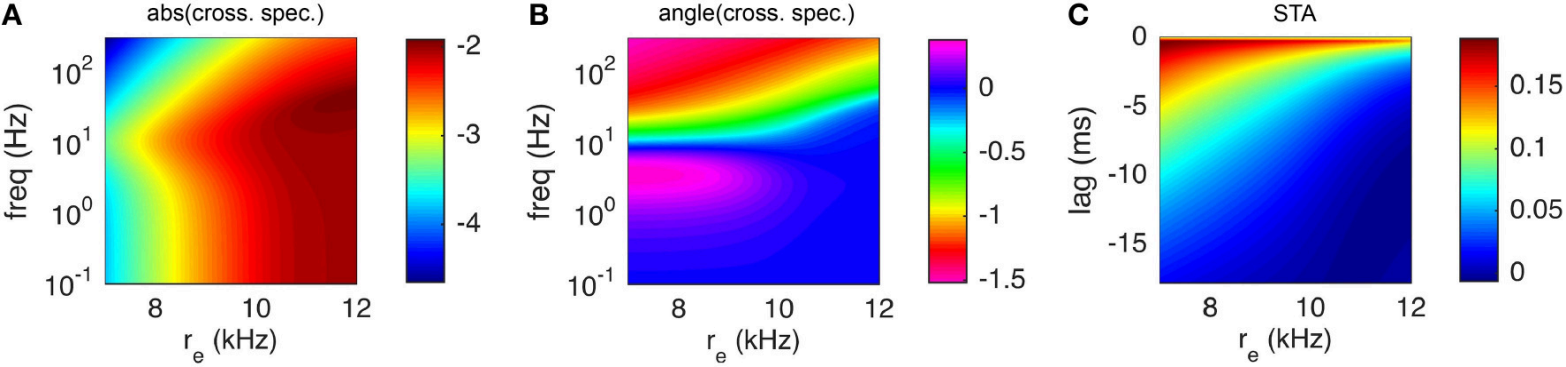
**Spike triggered average input current and cross-spectral density under a white noise perturbation as a function of presynaptic excitatory rate**. Same as Figure 8 except that only numerical results are shown, using the matched variance approximation, over a range of values of the excitatory presynaptic rate, *r*_*e*_.

A comparison of the numerically computed values of *STA*(τ) and ${\tilde{C}}_{us}\left(f\right)$ to their estimates from Monte-Carlo simulations of the full AdEx model shows good agreement (Figure 8). Note that, since the cross-spectral density is proportional to the susceptibility, Figure 8 also reveals the shape of the susceptibility function and demonstrates the interpolation used (compare coarse mesh indicated by blue circles to interpolated fine mesh indicated by solid blue curve). The peak in the susceptibility function arises from resonance inherited by the spike- and voltage-based adaptation rules (Brunel et al., 2003; Richardson et al., 2003; Richardson, 2009; Ocker and Doiron, 2014). This resonance is captured by the numerical approximation because the fixed point computation of the susceptibility function takes into account the linear filtering of the adaptation currents (see Appendix D) as in Richardson (2009) and Ocker and Doiron (2014). Computing the numerical approximation to the cross-spectral density and spike triggered average over a larger range of presynaptic excitatory rates, *r*_*e*_ shows that this peak is a robust feature of the adaptive model at various firing rates (Figure 9).

Numerical computation of the linear response function, cross-spectrum and spike triggered average was extremely efficient, with all three computed in 21 ms on average for each value of *r*_*e*_ used in Figure 9. As noted above, this efficiency was obtained by first computing *S*_*r*_(*f*) at a coarse and non-uniform mesh of frequencies, then using polynomial interpolation to approximate the response at a fine, uniform grid of frequencies so that the inverse Fast Fourier Transform can be used to calculate *STA*(τ). Using this approach, the threshold-integration scheme was only called 30 times (one for each coarse frequency mesh point), but capturing the fast and slow timescales in *STA*(τ) required interpolating these 30 values to a fine mesh of 20,001 frequency values (0 to 2 kHz with 0.1 Hz steps). To estimate the efficiency added by our interpolation scheme, we tried computing the susceptibility directly on the finer mesh (with *r*_*e*_ = 10 kHz). This required 20,001 calls to the threshold-integration scheme and took 2.5 s. Thus, our interpolation approach is about 100 times faster than computing the susceptibility at each frequency directly.

### 3.5. Computing the cross-covariance function between the spiking response of two model neurons

We now consider two identical versions of the pyramidal neuron model, each identical to the individual models considered above, but with the additional assumption that a proportion *c* = 0.1 of the excitatory presynaptic spike times are shared between the neurons. This scenario models two neurons with overlapping presynaptic pools. These shared presynaptic spikes introduce correlations between the inputs to the two neurons, which in turn introduces correlations between their postsynaptic spike trains,

$$s_{k}(t)=\sum_{j}\delta (t-t_{k,j})$$

where *t*_*k, j*_ is the *j*th spike of neuron *k* = 1, 2.

Linear response theory can be used to show that the cross-spectral density is given to first order in *c* by Lindner et al. (2005), de la Rocha et al. (2007), and Shea-Brown et al. (2008)

$${\tilde{C}}_{s_{1}s_{2}}(f)=|S_{r}(f){|}^{2}{\tilde{C}}_{I_{1}I_{2}}(f)$$

where *S*_*r*_(*f*) is the susceptibility function of both neurons and

$${\tilde{C}}_{I_{1}I_{2}}(f)=cJ_{\text{e}}^{2}r_{\text{e}}$$

is the cross-spectral density between the neurons' input currents. The cross-covariance can then be computed through an inverse Fourier transform of the cross-spectral density (see Methods).

**Figure 10:**
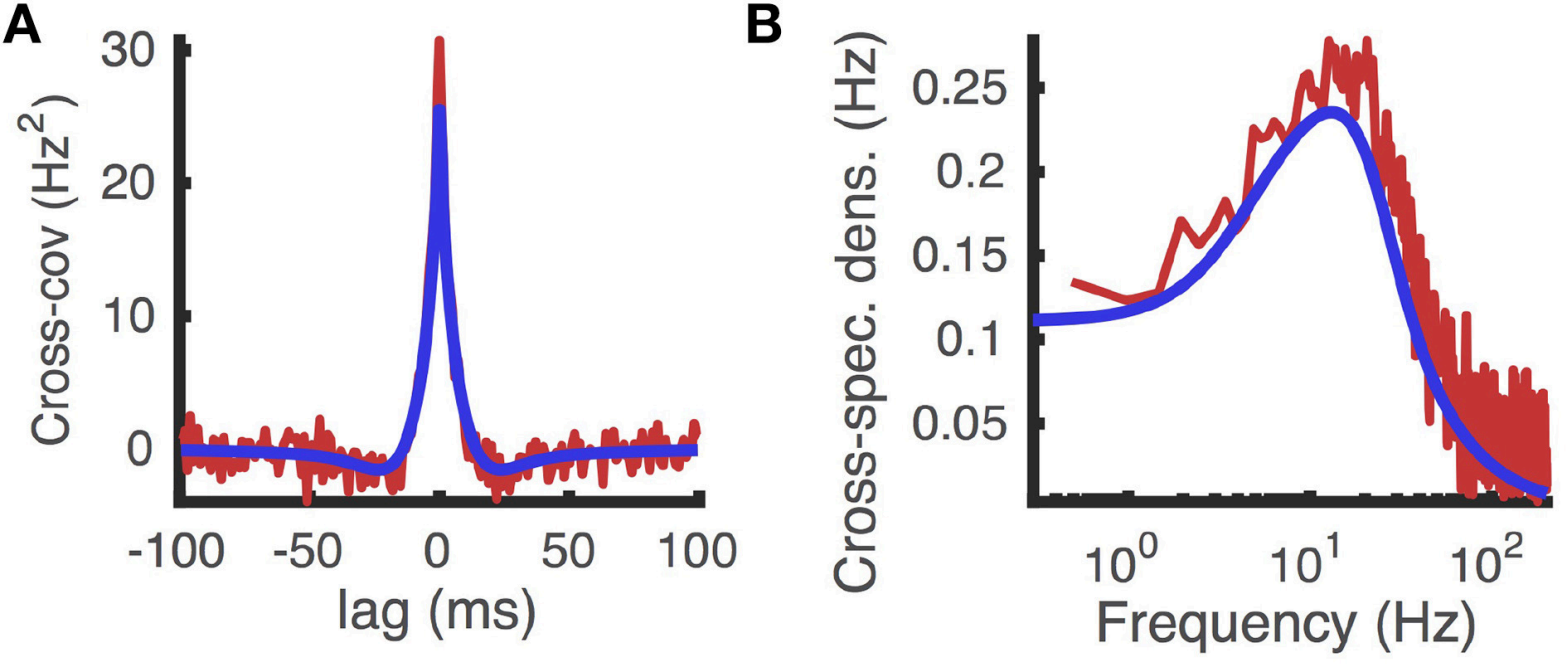
**Cross-covariance between two identical neurons receiving shared excitatory synaptic input**. **(A)** Cross-covariance and **(B)** cross-spectral density between the spike trains produced by two identical AdEx model neurons that share 10% of their excitatory presynaptic spike times, which occur as a homogeneous Poisson process. Computed from Monte Carlo simulations (red) and from the matched variance approximation (blue). The temporally uncorrelated input model was used. All parameters are as in Table 1.

Comparing the cross-covariance and cross-spectral density approximated numerically using this method to those computed from Monte-Carlo simulations shows reasonable agreement (Figure 10). Moreover, the numerical approximations were computed efficiently, taking less than 50 ms.

An analogous approach can be applied when the neurons share inhibitory presynaptic inputs too. Moreover, an extension of this method can be applied to compute the correlation between synaptically coupled neurons and the entire matrix of cross-correlations in large, sparsely coupled networks (Ostojic et al., 2009; Trousdale et al., 2012). Thus, our numerical methods can be used to efficiently approximate the correlation structure of large populations of synaptically coupled adaptive model neurons.

### 3.6. A matched variance approximation with temporally correlated input currents

So far we have considered numerical approximations obtained for a model of temporally uncorrelated synaptic input currents (see Methods). Synaptic currents *in vivo* are temporally correlated due to synaptic kinetics and temporal correlations in presynaptic spike timing. This shortcoming has been partially overcome by approximations that scale the diffusion coefficient to capture the steady-state free membrane potential variance of the full model (Alijani and Richardson, 2011; Hertäg et al., 2014), analogous to the matched variance approximation to adaptation dynamics described above. In these previous approaches, the approximation was only applied to synaptic input currents with an exponentially-shaped auto-correlation function, which can be used to model Poisson presynaptic spiking with exponential post-synaptic current waveforms. Moreover, these previous approaches were either applied to non-adaptive neuron models (Alijani and Richardson, 2011) or did not account for the effects of sub-threshold adaptation on the free membrane potential variance (Hertäg et al., 2014). We next extend this approach to account for sub-threshold adaptation and arbitrary stationary stochastic input.

First note that the linearity of Equations (15) allows the steady-state variance of *U*(*t*) to be computed for any stationary input *I*(*t*). Specifically, note that each line in Equation (15) implements a linear filter so that, for sufficiently large *t*,

(20) $$\begin{array}{l} U(t)=(K_{v}*I)(t)-(K_{v}*W)(t)+c_{1}(t)\\ W(t)=a(K_{w}*U)(t)+c_{2}(t) \end{array}$$

where *c*_1_(*t*) and *c*_2_(*t*) are asymptotically constant as *t* → ∞ and * denotes convolution. The kernels of the membrane potential and adaptation filters are given by

$$K_{v}(t)=\frac{1}{C_{m}}e^{-t/\tau_{m}}\Theta (t),$$

and

$$K_{w}(t)=\frac{1}{\tau_{w}}e^{-t/\tau_{w}}\Theta (t),$$

where τ_*m*_ = *C*_*m*_∕*g*_*L*_ and Θ(*t*) is the Heaviside step function. Transitioning to the Fourier domain and using Equation (6), we can write Equations (20) as

$$\begin{array}{l} {\tilde{U}}_{T}(f)={\tilde{K}}_{v}(f){\tilde{I}}_{T}(f)-{\tilde{K}}_{v}(f){\tilde{W}}_{T}(f)\\ {\tilde{W}}_{T}(f)=a{\tilde{K}}_{w}(f){\tilde{U}}_{T}(f) \end{array}$$

for sufficiently large *T* where

$${\tilde{K}}_{v}(f)=\frac{\tau_{m}}{C_{m}(1+2\pi if\tau_{m})}$$

and

$${\tilde{K}}_{w}(f)=\frac{1}{1+2\pi if\tau_{w}}$$

are the Fourier transforms of *K*_*v*_(*t*) and *K*_*w*_(*t*). Here, ${\tilde{U}}_{T}\left(f\right)$, ${\tilde{I}}_{T}\left(f\right)$ and ${\tilde{w}}_{T}\left(f\right)$ are defined by Equation (5). This is a linear system of algebraic equations that is easily solved at each frequency, *f*, to obtain

$${\tilde{U}}_{T}(f)=\frac{{\tilde{K}}_{v}(f)}{1+a{\tilde{K}}_{v}(f){\tilde{K}}_{w}(f)}{\tilde{I}}_{T}(f).$$

The power spectral density of *U*(*t*) is therefore given by (see Equation 7)

$$\begin{array}{l} {\tilde{C}}_{UU}(f)=\underset{T\to \infty }{\lim}|{\tilde{U}}_{T}(f){|}^{2}\\ \;=\frac{|{\tilde{K}}_{v}(f){|}^{2}}{|1+a{\tilde{K}}_{v}(f){\tilde{K}}_{w}(f){|}^{2}}{\tilde{C}}_{II}(f) \end{array}$$

where ${\tilde{C}}_{II}\left(f\right)$ is the power spectral density of *I*(*t*). The steady-state variance of the free membrane potential is then given by

$$\text{var}(U)=\int_{-\infty }^{\infty }{\tilde{C}}_{II}(f)|K_{\text{eff}}(f){|}^{2}df.$$

This expression is valid for any stationary input, *I*(*t*), for which ${\tilde{C}}_{II}\left(f\right)$ is defined and well-behaved (Yaglom, 2004).

Under the diffusion approximation in Equation (17), the free membrane potential variance is given by Equation (18). Combining these expressions shows that the diffusion approximation accurately captures the free membrane potential variance by taking

(21) $$D_{\text{eff}}=\frac{C_{m}^{2}}{\tau_{m}}\int_{-\infty }^{\infty }{\tilde{C}}_{II}(f)|K_{\text{eff}}(f){|}^{2}df$$

which defines the matched variance approximation to temporally correlated inputs. In the absence of voltage-based adaptation (*a* = 0) and when *I*(*t*) has an exponential-shaped auto-covariance function, this approximation is identical to the ones in Alijani and Richardson (2011) and Hertäg et al. (2014).

We consider an input model where temporal correlations are introduced by synaptic kinetics and by temporal correlations in the timing of presynaptic spikes (see Methods). Under this input model, the power spectral density of the synaptic input current is given by Rosenbaum et al. (2012b)

$$\begin{array}{l} {\tilde{C}}_{II}(f)=\;J_{\text{e}}^{2}[r_{\text{e}}+{\tilde{C}}_{\nu \nu }(f)]{\tilde{\alpha }}_{\text{e}}(f)\\ \;+J_{\text{i}}^{2}[r_{\text{i}}+{\tilde{C}}_{\nu \nu }(f)]{\tilde{\alpha }}_{\text{i}}(f) \end{array}$$

where

$${\tilde{\alpha }}_{\text{e}}(f)=\frac{1}{1+4\pi^{2}\tau_{\text{e}}^{2}f^{2}}$$

is the Fourier transform of the excitatory postsynaptic current waveform,

$${\tilde{\alpha }}_{\text{i}}(f)=\frac{1}{\left(1+4\pi^{2}\tau_{r,\text{i}}^{2}f^{2})(1+4\pi^{2}\tau_{d,\text{i}}^{2}f^{2}\right)}$$

is the Fourier transform of the excitatory postsynaptic current waveform and

$${\tilde{C}}_{\nu \nu }(f)=\frac{2\sigma_{\nu }^{2}\tau_{\nu }}{1+4\pi^{2}\tau_{\nu }^{2}f^{2}}$$

is the power spectral density of the firing rate fluctuations, ν(*t*). Under this model, the integral in Equation (21) can be approximated numerically (we used the integral command in Matlab) to obtain the corrected diffusion coefficient that defines the matched variance approximation.

**Figure 11:**
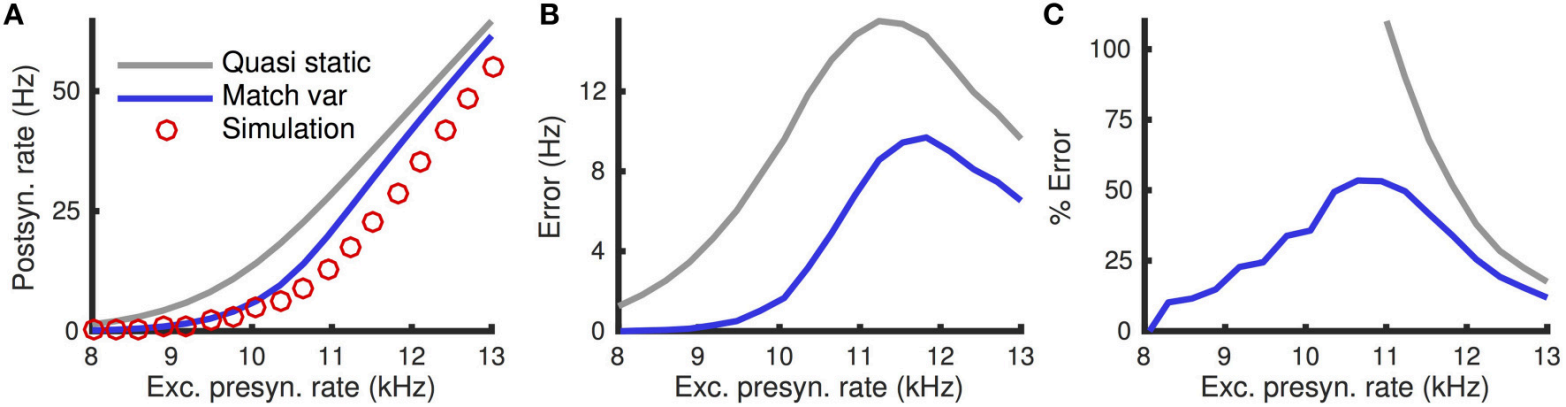
**Quasi-static and matched variance approximations to firing rates when input currents are temporally correlated**. **(A)** Steady-state postsynaptic Firing rate, *r*_0_, as the presynaptic excitatory rate, *r*_*e*_, increases. Plotted for Monte-Carlo simulations (red circles), quasi-static approximation (gray curve) and matched variance approximation (blue curve) for the temporally correlated input model (see Methods). **(B,C)** Error and % error of the quasi-static and matched variance approximations compared to Monte-Carlo simulations. The temporally correlated input model was used. All parameters other than *r*_*e*_ are as in Table 1.

Testing this matched variance approximation against Monte-Carlo simulations shows that it captures the overall shape of the dependence of firing rate on presynaptic excitatory rate (Figure 11A, compare blue and red), but introduces large errors (Figures 11B,C blue). Nevertheless, it substantially out-performs the quasi-static approximation obtained using the diffusion coefficient in Equation (10) (Figures 11B,C gray).

The timescale of temporal input correlations is determined by the timescale of firing rate fluctuations, τ_ν_, as well as the timescales of synaptic kinetics, τ_*e*_, τ_*r, i*_ and τ_*d, i*_. In the limit that all of these timescales approach zero, the input is approximated accurately by the temporally uncorrelated model. Thus, we expect the matched variance approximation for temporally correlated inputs to be approximately as accurate as for temporally uncorrelated inputs when these timescales are small.

**Figure 12:**
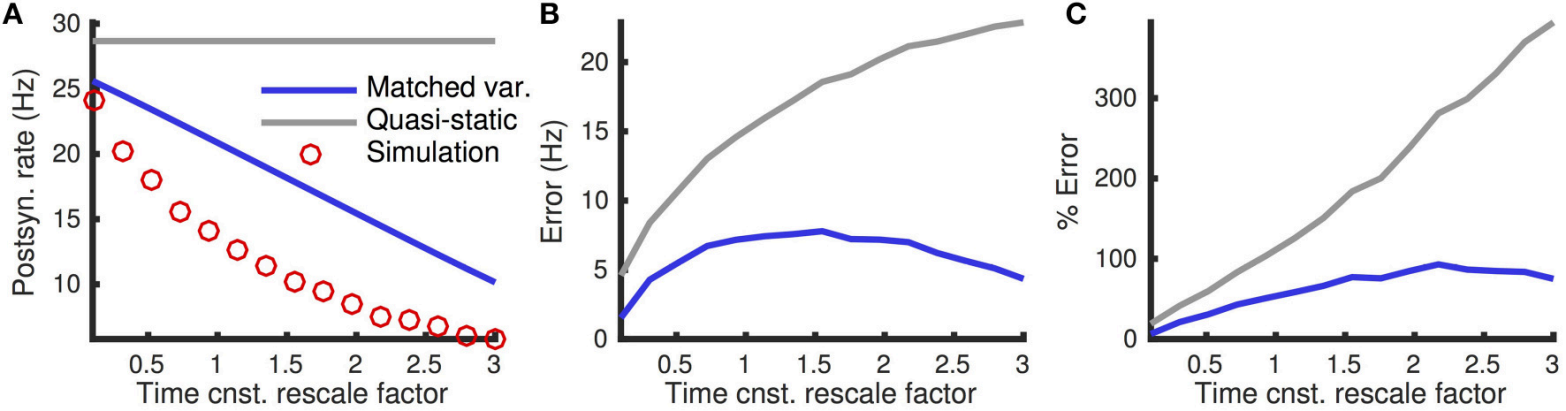
**Firing rates and errors as a function of temporally correlated input timescale**. **(A)** Steady state firing rate from simulations (red circles), quasi-static approximation (gray) and matched variance approximation (blue) as the timescales (τ_*e*_, τ_*r, i*_, τ_*d, i*_ and τ_ν_) are scaled by different factors. **(B,C)** Errors and percent errors from **(A)**. The excitatory presynaptic rate was increased to *r*_*e*_ = 11 kHz and the time bin width was decreased to *dt* = 0.05 for simulations. The temporally correlated input model was used. All parameters other than *r*_*e*_, τ_*e*_, τ_*r, i*_, τ_*d, i*_ and τ_ν_ are as in Table 1.

To test this prediction, we compared the matched variance (and quasi-static) approximation to Monte-Carlo simulations as the input timescales were scaled (Figure 12). As expected, the matched variance approximation performs well when input timescales are fast, but not when they are slow (Figure 12, blue curves). The quasi-static approximation, obtained using the diffusion coefficient in Equation (10), does not capture any dependence of the firing rate on the input timescales and performs worse than the matched variance approximation (Figure 12, compare blue and gray curves).

These examples demonstrate that the matched variance approximation offers an improvement over approximations that do not correct for synaptic timescales at all, but there is substantial room for improvement in the approximation of firing rates of neuron models drive by temporally correlated inputs.

## 4. Discussion

We derived and tested a suite of accurate and efficient numerical methods for approximating the response statistics of the adaptive integrate-and-fire neuron models with noisy synaptic input. We introduced an extension of the matched variance approximation (Alijani and Richardson, 2011; Hertäg et al., 2014) to capture the effects of sub-threshold, voltage-based adaptation currents on passive membrane variability. This approximation is more accurate than previously posed quasi-static approximations to the effects of adaptation (Richardson, 2009; Ocker and Doiron, 2014), especially when voltage-based adaptation currents are strong and/or fast.

Our approximation is also applicable in the case of arbitrary, temporally correlated synaptic inputs. This extends previous approximations, which are applicable only when inputs have exponentially-shaped auto-correlations and which do not account for the effects of voltage-based adaptation currents on sub-threshold membrane potential variability (Alijani and Richardson, 2011; Hertäg et al., 2014). In the examples we considered, however, the matched variance approximation performed relatively poorly when strong adaptation was combined with temporally correlated inputs (Figures 11, 12).

The methods presented here rely heavily on previously developed techniques. The threshold-integration method was developed previously (Richardson, 2007, 2008) and we only improved the computational efficiency. Similarly, the fixed point iteration method for approximating the effect of an adaptation current was considered previously (Richardson, 2009; Ocker and Doiron, 2014) and we derived a re-scaled diffusion coefficient to account for variability in sub-threshold adaptation currents. A matched variance approximation has been developed to account for temporally correlated synaptic inputs and we extended this method to account for sub-threshold adaptation currents.

The response of model neurons to temporally correlated inputs has also been approximated using timescale separation when synaptic kinetics are much slower or faster than membrane dynamics (Moreno-Bote and Parga, 2006). However, with the exception of NMDA synaptic kinetics, synaptic and membrane timescales in cortex are not so separated, especially when one accounts for faster “effective” membrane time constant arising from the barrage of conductance-based inputs received by neurons *in vivo* (Destexhe and Paré, 1999; Destexhe et al., 2003; Kuhn et al., 2004).

Computational efficiency is often ignored when reporting on numerical methods for stochastic neuron models. We carefully designed our methods to be computationally efficient and tested this efficiency. As a consequence, our methods allow the computation of membrane potential response properties orders of magnitude faster than some previous methods for which timing has been reported (Hertäg et al., 2014), though our methods are potentially less accurate. While the difference between milliseconds and seconds of computation time is unimportant when computing the firing rate of a single neuron model for one set of parameters, it could be critical for applications where response properties need to be computed many times. For example, computing rates and correlations in a large recurrent network requires solving a fixed point point problem in which stationary rates and susceptibility functions are computed many times per neuron (Amit and Brunel, 1997; Brunel and Hakim, 1999; Trousdale et al., 2012). Additionally, fitting a network model to recorded multicellular data can require the repeated evaluation of rates and susceptibility functions for several parameter values and several neurons (Pernice and Rotter, 2013).

Our methods compromise some accuracy for biological realism. Specifically, the inclusion of temporally correlated noise and adaptation currents forced us to use approximations that introduced errors in the computation of response statistics. Real neurons receive temporally correlated input and have adaptation currents. Thus, the inclusion of these complicating factors is a necessity for realistic modeling. We hope that future work can refine the approximations discussed here to reduce their errors further.

One shortcoming of our approach is that we used a highly simplified synaptic input model. We only considered synaptic timescales modeled after AMPA and GABA_*B*_ mediated kinetics. NMDA mediated kinetics are much slower and are best captured by a conductance-based synapse model that depends non-linearly on voltage (Dayan and Abbott, 2001). Thus, we expect our approximations, which rely on a current-based synapse model with fast kinetics, to perform poorly for NMDA mediated synaptic kinetics. The matched variance approximation be extended to conductance-based synapse models, using approximations for the free membrane potential variance for such a models (Rudolph and Destexhe, 2003; Richardson, 2004; Richardson and Gerstner, 2005; Lindner and Longtin, 2006).

Another shortcoming of our approach is that we were unable to accurately approximate the power spectral density, auto-correlation or Fano factor of the postsynaptic spike trains. The inability to compute temporal spike train statistics is primarily due to the non-renewal properties introduced by the strong and slow adaptation current as well as temporally correlated synaptic input. Methods have been developed for approximating the spiking statistics of integrate-and-fire models with adaptation currents and temporally correlated inputs (Middleton et al., 2003; Lindner, 2004; Moreno-Bote and Parga, 2006; Ly and Tranchina, 2009; Richardson and Swarbrick, 2010; Dummer et al., 2014; Schwalger et al., 2015; Shiau et al., 2015). It would be interesting to investigate whether some of these methods could be integrated with the methods developed here.

We used a linear model of adaptation in the sense that the dependence of *V*′(*t*) on *w* in Equation (1) and the dependence of *w*′(*t*) on *V* in Equation (2) is linear. This linearity was used in the matched variance approximation because it allowed the variance of the free membrane potential to be computed in closed form in the presence of sub-threshold adaptation. The quasi-static approximation has been applied to models in which the adaptation variable depends nonlinearly on *V* (Richardson, 2009). Extending the matched variance approach to nonlinear adaptation would be a challenge since it would require the computation of the free membrane potential variance in a nonlinear system of stochastic differential equations.

Another approach to accounting for adaptation currents would be to solve the full two-dimensional Fokker-Planck equation for the bivariate density of *w*(*t*) and *V*(*t*). Analytical approaches have been developed for spike-based adaptation when noise is weak and spiking is mean-driven (Schwalger and Lindner, 2015). Numerical solutions to the general problem are difficult because threshold-integration cannot be applied directly to the two-dimensional equation. Instead, one could use a numerical scheme such as finite difference, finite volume or finite element methods (see a similar approach for a two-neuron model in Rosenbaum et al., 2012a). Our future work will consider these approaches.

## Author contributions

The author confirms being the sole contributor of this work and approved it for publication.

### Conflict of interest statement

The author declares that the research was conducted in the absence of any commercial or financial relationships that could be construed as a potential conflict of interest.

## A. Appendix

### A. Steady state Fokker-Planck equation and numerical scheme for its solution

The stochastic differential equation in Equation (13) can be re-written in the form

$$\frac{dV}{dt}=A_{0}(V)+\sqrt{2D_{0}(V)}\eta (t)$$

where η(*t*) is Gaussian white noise,

$$A_{0}(V)=\;C_{m}^{-1}(-g_{L}(V-E_{L})+\psi (V)-\mu_{w}+\mu_{I})$$

is the drift coefficient and

$$D_{0}=\frac{D_{I}}{C_{m}^{2}}$$

is the diffusion coefficient. The matched variance approximation is identical except the diffusion coefficient from the input current, *D*_*I*_, is replaced by an effective diffusion coefficient, *D*_*eff*_, that accounts for the fluctuating adaptation current. The Fokker-Planck equation for the stationary probability density, *P*_0_(*v*), of the diffusion process, *V*(*t*), can be written in the form of a continuity equation (Risken, 1996; Richardson, 2007, 2008),

$$\frac{d}{dv}J_{0}(v)=r_{0}\delta (v-V_{\text{re}})$$

where

$$J_{0}(v)=A_{0}(v)P_{0}(v)-D_{0}\frac{dP_{0}(v)}{dv}$$

is the stationary probability flux and *r*_0_ = *J*_0_(*V*_*th*_) is the stationary firing rate of the neuron. The absorbing boundary at threshold imposes the Dirichlet boundary condition, *P*_0_(*V*_*th*_) = 0. An additional condition is given by noting that *P*_0_(*v*) is a probability density so that

$$\int_{-\infty }^{V_{\text{th}}}P_{0}(v)dv=1.$$

We now describe a numerical method for computing the steady state statistics of the AdEx model with the adaptation current, *w*(*t*), replaced by its steady-state mean, 〈*w*〉. This is identical to solving a non-adaptive EIF model with an adjusted input current, *I*(*t*) → *I*(*t*) − 〈*w*〉. An identical method was used in Richardson (2007, 2008) except that a midpoint rule was used to approximate integrals whereas we use Simpson's rule.

The steady state probability flux is defined by

$$J_{0}(v)=A_{0}(v)P_{0}(v)-D_{0}\frac{dP_{0}(v)}{dv}$$

which satisfies the trivial differential equation

$$J_{0}^{\prime }(v)=r_{0}\delta (v-V_{\text{re}})$$

with boundary condition *J*_0_(*V*_*th*_) = *r*_0_ where *r*_0_ is the stationary firing rate. Recall also that *P*_0_(*V*_*th*_) = 0. Now define *p*_0_(*v*) = *P*_0_(*v*)∕*r*_0_ and *j*_0_(*v*) = *J*_0_(*v*)∕*r*_0_. We can then re-write the Fokker-Planck equation as a system,

$$\begin{array}{l} p_{0}^{\prime }(v)=\frac{1}{D_{0}}\left[A_{0}(v)p_{0}(v)-j_{0}(v)\right]\\ j_{0}^{\prime }(v)=\delta (v-V_{\text{re}}) \end{array}$$

with boundary conditions *j*_0_(*V*_*th*_) = 1 and *p*_0_(*V*_*th*_) = 0. The solution for *j*_0_(*v*) is given trivially as

$$j_{0}(v)=\{\begin{array}{l} 1\;v\geq V_{\text{re}}\\ 0\;v\leq V_{\text{re}} \end{array}$$

and the solution for *p*_0_(*v*) can be found numerically by integrating backwards from *v* = *V*_*th*_. Specifically, we re-write the equation for *p*_0_(*v*) as

$$-p_{0}^{\prime }(v)=G(v)p_{0}(v)+H(v)$$

where *G*(*v*) = −*A*_0_(*v*)∕*D*_0_(*v*) and *H*(*v*) = *j*_0_(*v*)∕*D*_0_. This linear differential equation can be solved numerically for the scaled density *p*_0_(*v*). Once *p*_0_ is found, the firing rate and the true density are given by

$$r_{0}={\left(\int_{V_{\text{lb}}}^{V_{\text{th}}}p_{0}(v)dv\right)}^{-1}$$

and *P*_0_(*v*) = *r*_0_*p*_0_(*v*).

To solve Equation (A2) numerically, we choose a lower boundary, *V*_*lb*_, sufficiently below *E*_*L*_ and *V*_*re*_ that the density is approximately zero there, then discretize the state space [*V*_*lb*_, *V*_*th*_] into a possibly non-uniform mesh ${\left\{v_{k}\right\}}_{k=1}^{n}$. We denote by $p_{0}^{k}$ the numerical approximation to *p*_0_(*v*_*k*_). Since the last element represents threshold, *v*_*n*_ = *V*_*th*_, the boundary conditions give us $p_{0}^{n}=0$ and $j_{0}^{n}=1$. Equation (A2) can then be integrated backwards from threshold using a numerical integration scheme.

The most straightforward integration scheme for Equation (A2) is a standard Euler scheme defined by setting

$$p_{0}^{k}=p_{0}^{k+1}+(p_{0}^{k+1}G_{k}+H_{k})\Delta v_{k}$$

where *G*_*k*_ and *H*_*k*_ represent the functions *G*(*v*) and *H*(*v*) evaluated at the midpoint (*v*_*k*_+*v*_*k*+1_)∕2 and where Δ*v*_*k*_ = *v*_*k*+1_−*v*_*k*_. This method is only accurate for very fine meshes, due in part to the large advection term for *v* > *V*_*T*_ (see Figure 4, green curve).

The “modified Euler” scheme proposed in Richardson (2007, 2008) takes advantage of the linearity of Equation (A2) to first write an analytical solution

$$p_{0}^{k}=p_{0}^{k+1}\text{exp}\left(\int_{v_{k}}^{v_{k+1}}Gdv\right)+\int_{v_{k}}^{v_{k+1}}H(u)\text{exp}\left(\int_{v_{k}}^{u}Gdv\right)du.$$

Using a midpoint rule to approximate the integrals in this expression gives an increment defined by

$$p_{0}^{k}=p_{0}^{k+1}\text{exp}\left(G_{k}\Delta v_{k}\right)+H_{k}\frac{\text{exp}\left(G_{k}\Delta v_{k}\right)-1}{G_{k}}.$$

For mesh points where *G*_*k*_ is approximately zero, numerical inaccuracies are circumvented by replacing the fraction in this expression with its limit,

$$\frac{\text{exp}\left(G_{k}\Delta v_{k}\right)-1}{G_{k}}\to \Delta v_{k}$$

as *G*_*k*_ → 0. This scheme gives better convergence results than the Euler scheme for coarse meshes, but not for finer meshes (Figure 4).

To improve the numerical accuracy over all mesh sizes, we propose a new scheme that uses the same analytical expression (A3), but approximates the integrals using Simpson's method instead of a midpoint method. This is achieved by taking the increment defined by

$$p_{0}^{k}=p_{0}^{k+1}\text{exp}\left(I_{1}^{k}\right)+I_{2}^{k}$$

where

$$I_{1}^{k}=\frac{1}{6}\left(G(v_{k})+4G_{k}+G(v_{k+1})\right)\Delta v_{k}$$

is the Simpson's rule approximation to the first integral in Equation (A3). Approximating the second integral is more difficult because it is a double integral. Also, *H*(*v*) = *j*_0_(*v*)∕*D*_0_ has a jump discontinuity inherited from *j*_0_(*v*). Since Simpson's method does not converge for integrals of discontinuous functions, we must evaluate *H*(*v*) at the midpoints only. Thus, the outer integral is approximated by

$$I_{2}^{k}=\frac{H_{k}}{6}\left(1+4\exp(I_{3}^{k})+\exp(I_{1}^{k})\right)\Delta v_{k}.$$

where $I_{3}^{k}$ is the Simpson's rule approximation to the integral of *G* from *v*_*k*_ to (*v*_*k*_+*v*_*k*+1_)∕2, which is given by

$$I_{3}^{k}=\frac{1}{6}\left(G(v_{k})+4G(0.75v_{k}+0.25v_{k+1})+G_{k}\right)\Delta v_{k}/2.$$

While this numerical scheme requires several more arithmetic operations per increment, it converges much more quickly than the other two methods, giving much more accurate results at coarser meshes using less CPU time (Figure 4). For all figures, except where indicated otherwise in figure caption, we set *V*_*lb*_ = −100 mV and used a mesh that was uniform with step size Δ*v* = 0.1 mV, except for the two mesh points surrounding *V*_*re*_ and all mesh points above *V*_*T*_ where the mesh size was refined to Δ*v*∕2 = 0.05 mV. This yielded 654 mesh points. For Figure 4, an analogous mesh was used, with Δ*v* scaled by different factors.

### B. Iterative method for computing fixed point of steady-state statistics

The steady-state mean adaptation current, μ_*w*_, can be computed from the steady-state rate, *r*_0_, and steady-state mean membrane potential, μ_*V*_, in closed form using Equation (12). Conversely, the steady state rate and mean membrane potential can be computed from the steady-state adaptation current by numerically solving the associated Fokker-Planck equation as described in Appendix A.1. To find the steady-state statistics, we used an iterative method to compute the fixed point of these mappings, similar to the approach in Richardson (2009) and Ocker and Doiron (2014).

Specifically, define

$$g(v,r)=a(v-E_{L})+\tau_{w}br.$$

to be the function determined by Equation (12) that maps mean membrane potential and firing rate to mean adaptation current. Similarly define *h*(*w*) = (*v, r*) to be the mapping from mean adaptation current to mean membrane potential and firing rate determined by numerically solving the Fokker-Planck equation. Choose an initial value *w*_0_ = 0, then iteratively calculate

$$\left(v_{j},r_{j})=h(w_{j-1}\right)$$

and

$$w_{j}=(1-q)w_{j-1}+qg(v_{j},r_{j})$$

until

$$\left|\frac{v_{j}-v_{j+1}}{v_{j}+v_{j+1}}\right|+\left|\frac{r_{j}-r_{j+1}}{r_{j}+r_{j+1}}\right|<\epsilon$$

for some small relative error tolerance, ϵ. We used ϵ = 10^−4^. Here, 0 < *q* ≤ 1 controls the size of the iterative step. Larger values of *q* can potentially yield faster convergence, but also make the iterations more susceptible to oscillations that do not converge. Smaller values of *q* converge slowly even in the best case, but are more robust. To obtain a robust and fast iterative method, we implemented a scheme that first searches for a fixed point using *q* = 0.25. If convergence is not realized within 50 iterations, *q* is successively decreased until convergence is obtained.

### C. Steady-state free membrane potential variance for temporally uncorrelated inputs

We now derive Equation (16) for the steady-state variance of the free membrane potential under the two-dimensional dynamics of Equation (15) and under the temporally uncorrelated input model. First note that, when *I*(*t*) is temporally uncorrelated, the first two moments of *U*(*t*) and *W*(*t*) are identical to the first two moments under the substitution $I\left(t\right)\to \mu_{I}+\sqrt{2D_{I}}\eta \left(t\right)$, which gives the two-dimensional stochastic differential equation

$$\frac{d\vec{X}}{dt}=-A\vec{X}+C+B\eta (t)$$

where η(*t*) is white noise,

$$\begin{array}{l} \vec{X}(t)=\left[\begin{array}{c} U(t)\\ W(t) \end{array}\right],\\ A=\left[\begin{array}{c} 1/\tau_{m}1/C_{m}\\ -a/\tau_{w}1/\tau_{w} \end{array}\right], \end{array}$$

$$B=\left[\begin{array}{c} \sqrt{2D_{I}}/C_{m}\\ 0 \end{array}\right]$$

and *C* is a constant 2 × 1 vector. Thus, $\vec{X}\left(t\right)$ is a two-dimensional Ornstein-Uhlenbeck process and the steady-state covariance matrix has a known closed form (Gardiner, 1985)

$$\Sigma =\frac{\det(A)BB^{T}+QBB^{T}Q^{T}}{2\text{Tr}(A)\det(A)}$$

where *Q* = *A* − *Tr*(*A*)*I*_2_ and *I*_2_ is the 2 × 2 identity matrix. Substituting *A* and *B* into this expression, the steady state variance of *U*(*t*) is given by the top-right element of the covariance matrix, *var*(*U*) = Σ(1, 1), which is easily simplified to give Equation (16).

### D. Numerical methods for computing the susceptibility and linear response functions

We first consider the response of the neuron to a periodic perturbation, which can be combined with linear response theory to calculate the response to more general forms of modulations (Risken, 1996; Fourcaud and Brunel, 2002). Consider a complex, oscillatory perturbation of the synaptic input of the form

$$I(t)=I_{0}(t)+\epsilon e^{2\pi ift}$$

where *I*_0_(*t*) is the un-perturbed input with stationary statistics considered above. Here, *f* is the frequency of the modulations and the small parameter ϵ scales the amplitude. Using a complex modulation allows the amplitude and phase shift of the response to be calculated simultaneously (Richardson, 2007, 2008).

When ϵ = 0, we recover the steady state model from Appendices A and B. To first order in ϵ, the time-dependent part of the statistics are periodic with frequency *f* after an aperiodic transient (Risken, 1996; Fourcaud and Brunel, 2002). Therefore, for sufficiently large *t* and to first order in ϵ, we can write

$$\begin{array}{l} P(v,t)=P_{0}(v)+\epsilon P_{1}(v)e^{2\pi ift}\\ \;r(t)=r_{0}+\epsilon S_{r}(f)e^{2\pi ift}\\ \;\langle V(t)\rangle =\mu_{V}+\epsilon S_{V}(t)e^{2\pi ift}\\ \;\langle w(t)\rangle =\mu_{w}+\epsilon S_{w}(f)e^{2\pi ift} \end{array}$$

where *P*_0_(*v*), *r*_0_, μ_*V*_ and μ_*w*_ are the steady state statistics from above obtained by setting ϵ = 0 and computed using the methods outline in Appendices A and B.

The terms, *P*_1_(*v*), *S*_*r*_(*f*), *S*_*V*_(*f*) and *S*_*w*_(*f*) are the susceptibility functions of the probability density, firing rate, mean membrane potential and mean adaptation current. We next derive approximations of these quantities by adapting previously used methods for models with voltage-gated adaptation currents (Richardson, 2009; Ocker and Doiron, 2014).

First note that, due to the linearity of Equation (2), the value of *S*_*w*_ can be computed exactly in terms of *S*_*V*_ and *S*_*r*_. This is accomplished by making the substitutions from Equation (A4) into Equation (11), collecting all time-dependent terms and solving the resulting equation to obtain

$$S_{w}=\frac{aS_{V}+\tau_{w}bS_{r}}{2\pi if\tau_{w}+1}.$$

We next note that substituting 〈*w*(*t*)〉 from Equation (A4) into the equation for *V*(*t*) is equivalent to a non-adaptive EIF model with input current $I\left(t\right)=I_{0}\left(t\right)-\mu_{w}+\epsilon \left(1-S_{w}\right)e^{2\pi ift}+O\left(\epsilon^{2}\right)$. With this substitution, the oscillation in of the firing rate and mean membrane potential are to first order in ϵ by

$$\begin{array}{l} S_{r}=(1-S_{w})S_{r}^{EIF}\\ S_{V}=(1-S_{w})S_{V}^{EIF} \end{array}$$

where $S_{r}^{EIF}\left(f\right)$ is the susceptibility of the firing rate and $S_{V}^{EIF}\left(f\right)$ of the mean membrane potential for the non-adaptive EIF model (obtained by setting *a* = *b* = 0 in Equations 1–5).

The EIF susceptibility functions, $S_{r}^{EIF}$ and $S_{V}^{EIF}$, can be computed at a given frequency, *f*, using the threshold integration developed in Richardson (2007, 2008). We again improved previous implementations of the threshold integration method by implementing the algorithm in C and using Simpson's rule to evaluate the resulting integrals, as we describe below. Note that the susceptibility functions must be evaluated with a bias term shifted by the steady state mean adaptation current, μ_*I*_ → μ_*I*_−μ_*w*_, which is computed using the steady state solver described above.

In summary, *S*_*V*_ and *S*_*r*_ can each be computed as a function of *S*_*w*_ using Equations (A6) and *S*_*w*_ can be computed as a function of *S*_*V*_ and *S*_*r*_ using Equation (A5). Similar to the steady state statistics, this yields a fixed point problem for the modulated statistics, *S*_*v*_, *S*_*r*_ and *S*_*w*_. This fixed point problem could be solved iteratively, as done for the fixed point statistics above, but a simpler and more efficient approach is given by noting that the relationships between *S*_*r*_, *S*_*V*_ and *S*_*w*_ are linear, unlike the nonlinear dependence of *r*_0_ and μ_*V*_ on μ_*w*_ for the steady state problem. Therefore, the fixed point values of *S*_*r*_, *S*_*V*_ and *S*_*w*_ are easily found in closed form by solving the linear system determined by Equations (A5) and (A6).

In summary, the susceptibility functions, *S*_*r*_(*f*), *S*_*V*_(*f*) and *S*_*w*_(*f*) are computed by first solving the steady-state problem for *r*_0_ and μ_*V*_ on μ_*w*_ as described above, then computing the EIF susceptibility functions, $S_{r}^{EIF}\left(f\right)$ and $S_{V}^{EIF}\left(f\right)$, using threshold integration, then solving the linear system determined by Equations (A5) and (A6).

We now describe our numerical scheme for computing the EIF susceptibility functions, $S_{r}^{EIF}\left(f\right)$ and $S_{V}^{EIF}\left(f\right)$, that are used to compute the susceptibility functions for the full adaptive model as described above.

We adapted the numerical “threshold-integration” scheme introduced in Richardson (2007, 2008) except that we use Simpson's rule in place of the midpoint rule to approximate integrals, as described below. We review this approach now. A more detailed description can be found in Richardson (2007, 2008). The modulated membrane potential, *V*(*t*), obeys a stochastic differential equation of the form

$$\frac{dV}{dt}=A(V,t)+\sqrt{2D_{0}}\eta (t)$$

with drift coefficient

$$A(V,t)=A_{0}(V)+\epsilon A_{1}e^{2\pi ift},$$

The time-independent drift and diffusion coefficients, *A*_0_(*V*) and *D*_0_, are the same as for the steady state equation considered above and the coefficient for the time-dependent drift is $A_{1}=C_{m}^{-1}.$ The Fokker-Planck equation for the time-dependent probability density, *P*(*v, t*), can be written as a continuity equation

$$\frac{\partial }{\partial v}J(v,t)+\frac{\partial }{\partial t}P(v,t)=r(t)\delta (v-V_{\text{re}})$$

with probability flux

$$J(v,t)=A(v,t)P(v,t)-D_{0}\frac{\partial }{\partial v}P(v,t)$$

If we make the substitution for *P*(*v, t*) from Equations (A4) and expand to linear order in ϵ, we recover the steady state Fokker-Planck Equation (A1) for *P*_0_(*v*) and derive a new continuity equation for *P*_1_(*v*), given by

$$\frac{d}{dv}J_{1}(v)=S_{r}\delta (v-V_{\text{re}})-2\pi ifP_{1}(v)$$

where

$$J_{1}=AP_{0}+A_{0}P_{1}-D_{0}\frac{d}{dv}P_{1}$$

is the linear order perturbation of the probability flux defined by

$$J(v,t)=J_{0}(v)+\epsilon J_{1}(v)e^{2\pi ift}+O(\epsilon^{2}).$$

Boundary conditions are *P*_1_(*V*_*th*_) = 0 and

$$\int_{-\infty }^{V_{\text{th}}}P_{1}(v)dv=0.$$

The modulation in the firing rate is given by *S*_*r*_ = *J*_1_(*V*_*th*_) and the modulation in the mean membrane potential is given by integrating *vP*_1_(*v*). Note that *P*_0_(*v*) appears in the equation for *P*_1_(*v*), so the steady state statistics must be computed first. The continuity equation can be rewritten as as system of ordinary differential equations

$$\begin{array}{l} \frac{dP_{1}}{dv}=\frac{1}{D_{0}}A_{0}P_{1}+\frac{1}{D_{0}}\left[A_{1}P_{0}-J_{1}\right]\\ \frac{dJ_{1}}{dv}=S_{r}\delta (v-V_{re})-2\pi ifP_{1} \end{array}$$

As for the steady state equation, we reformulate the problem by defining rescaled density and flux functions. First decompose *P*_1_(*v*) and *J*_1_(*v*) as *P*_1_(*v*) = *S*_*r*_*p*_*r*_(*v*)+*p*_1_(*v*) and *J*_1_(*v*) = *S*_*r*_*j*_*r*_(*v*) + *j*_1_(*v*). The components satisfy the two systems of equations

$$\begin{array}{l} -p_{1}^{\prime }(v)=-\frac{1}{D_{0}}A_{0}p_{1}+\frac{1}{D_{0}}\left[j_{1}-A_{1}P_{0}\right]\\ -j_{1}^{\prime }(v)=2\pi ifp_{1} \end{array}$$

and

$$\begin{array}{l} -p_{r}^{\prime }(v)=-\frac{1}{D_{0}}A_{0}p_{r}+\frac{1}{D_{0}}j_{r}\\ -j_{r}^{\prime }(v)=2\pi ifp_{r}-\delta (v-V_{\text{re}}) \end{array}$$

with boundary conditions *p*_*r*_(*V*_*th*_) = *p*_1_(*V*_*th*_) = *j*_1_(*V*_*th*_) = 0 and *j*_*r*_(*V*_*th*_) = 1. As for the steady state equation for *p*_0_(*v*) considered above, these equations for *p*_1_(*v*) and *p*_*r*_(*v*) can be written as −*p*′(*v*) = *G*(*v*)*p*(*v*) + *H*(*v*). To integrate these equations, we use the same modified Euler method with Simpson's rule that we used for the steady state density (see Appendix A.1). As above, we use a forward Euler step to integrate the equations for *j*_1_ and *j*_*r*_. Once *p*_1_, *j*_1_, *p*_*r*_ and *j*_*r*_ have been found, the modulation in the firing rate can be found by noting that *J*_1_(*V*_*lb*_) ≈ 0 assuming that *V*_*lb*_ was chosen sufficiently below *E*_*L*_ and *E*_*i*_. Thus, we have

$$S_{r}=-\frac{j_{1}(V_{\text{lb}})}{j_{r}(V_{\text{lb}})}.$$

The modulation in *V*(*t*) can be found by noting that

$$S_{V}=\int_{V_{\text{lb}}}^{V_{\text{th}}}vP_{1}(v)dv=\int_{V_{\text{lb}}}^{V_{\text{th}}}v[r_{1}p_{r}(v)+p_{1}(v)]dv.$$
